# Bucket Fuser: Statistical Signal Extraction for 1D ^1^H NMR Metabolomic Data

**DOI:** 10.3390/metabo12090812

**Published:** 2022-08-29

**Authors:** Michael Altenbuchinger, Henry Berndt, Robin Kosch, Iris Lang, Jürgen Dönitz, Peter J. Oefner, Wolfram Gronwald, Helena U. Zacharias, Investigators GCKD Study

**Affiliations:** 1 Department of Medical Bioinformatics, University Medical Center Göttingen, 37077 Göttingen, Germany; 2 Department of Internal Medicine I, University Medical Center Schleswig-Holstein, Campus Kiel, 24105 Kiel, Germany; 3 Institute of Clinical Molecular Biology, Kiel University and University Medical Center Schleswig-Holstein, Campus Kiel, 24105 Kiel, Germany; 4 Institute of Functional Genomics, University of Regensburg, 93053 Regensburg, Germany; 5 Peter L. Reichertz Institute for Medical Informatics of TU Braunschweig and Hannover Medical School, 30625 Hannover, Germany

**Keywords:** NMR metabolomics, data preprocessing, feature extraction

## Abstract

Untargeted metabolomics is a promising tool for identifying novel disease biomarkers and unraveling underlying pathomechanisms. Nuclear magnetic resonance (NMR) spectroscopy is particularly suited for large-scale untargeted metabolomics studies due to its high reproducibility and cost effectiveness. Here, one-dimensional (1D) ^1^H NMR experiments offer good sensitivity at reasonable measurement times. Their subsequent data analysis requires sophisticated data preprocessing steps, including the extraction of NMR features corresponding to specific metabolites. We developed a novel 1D NMR feature extraction procedure, called Bucket Fuser (BF), which is based on a regularized regression framework with fused group LASSO terms. The performance of the BF procedure was demonstrated using three independent NMR datasets and was benchmarked against existing state-of-the-art NMR feature extraction methods. BF dynamically constructs NMR metabolite features, the widths of which can be adjusted via a regularization parameter. BF consistently improved metabolite signal extraction, as demonstrated by our correlation analyses with absolutely quantified metabolites. It also yielded a higher proportion of statistically significant metabolite features in our differential metabolite analyses. The BF algorithm is computationally efficient and it can deal with small sample sizes. In summary, the Bucket Fuser algorithm, which is available as a supplementary python code, facilitates the fast and dynamic extraction of 1D NMR signals for the improved detection of metabolic biomarkers.

## 1. Introduction

Untargeted metabolomics, which is the comprehensive study of all metabolites that are detectable in one biological specimen, is a promising tool for identifying novel disease biomarkers and gaining deeper insights into underlying disease pathomechanisms. The two main analytical methods that are employed for untargeted metabolomics are hyphenated mass spectrometry and nuclear magnetic resonance (NMR) spectroscopy. The latter approach is particularly suited for large-scale studies that involve hundreds to thousands of samples due to its high reproducibility across measurement time and instruments, minimal sample pretreatment processes, and cost effectiveness. Here, one-dimensional (1D) ^1^H NMR experiments, which simultaneously detect all proton-containing metabolites present at sufficient concentrations in a sample, offer good sensitivity at reasonable measurement times. Numerous studies have already demonstrated the ability of 1D ^1^H NMR to reveal novel biomarkers, e.g., in the context of kidney [1,2,3,4] and heart diseases [5,6], as well as all-cause mortality [7,8].

However, the subsequent analysis of 1D ^1^H NMR spectra requires sophisticated data preprocessing strategies. Prior to any statistical evaluation, NMR signals that correspond to specific metabolites need to be extracted from the spectra. Each extracted NMR signal or feature should ideally represent the same metabolite across the complete sample cohort. This requirement, which is of paramount importance for subsequent statistical and bioinformatic data analyses, is challenged by the fact that NMR signal positions can vary across specimens due to differences in sample pH, ionic strength, and measurement temperature, as well as metabolite–protein interactions. Potentially the most popular NMR feature extraction method that is used to compensate for NMR signal shifts across sets of spectra is equidistant bucketing or binning. Each spectrum is split into buckets/bins of equal size and the signals within each bucket are summed or integrated. This method is able to substantially reduce the high dimensionality of 1D ^1^H NMR spectral data and can thus lower both the burden of multiple tests and the problem of overfitting in the subsequent statistical hypothesis testing and machine learning data analysis. However, this method is not able to resolve strongly overlapping NMR signals in crowded regions, which are typically present in the 1D ^1^H NMR spectra of complex biofluids, such as urine or plasma. Over recent years, several more sophisticated methods for 1D ^1^H NMR metabolic feature extraction have been proposed, including Gaussian binning [9], adaptive binning [10], adaptive intelligent binning [11], and dynamic adaptive binning [12], as well as the statistical recoupling of variables (SRV) [13] and the pJRES binning algorithm (JBA) [14]. Especially the latter two approaches, which perform clustering of adjacent spectral regions based on covariance to correlation ratios, are computationally feasible even in the case of large metabolomics data sets [14].

In this study, we developed the Bucket Fuser (BF) algorithm, which is a novel 1D ^1^H NMR feature extraction procedure that is based on a regularized regression framework, which uses fused group Least-Absolute Shrinkage and Selection (LASSO) terms. BF dynamically constructs NMR features, which predominantly comprise the same NMR signals across one dataset. We demonstrated its performance using three different NMR datasets and benchmarked it against existing state-of-the-art NMR feature extraction methods, including equidistant binning, SRV, and JBA. Extensive performance evaluations were carried out via hypothesis testing and correlation analyses using absolutely quantified metabolite concentrations, including a thorough investigation of sample size dependence. The BF algorithm is freely available as a python implementation.

## 2. Methods

Let *Y* be a n×p matrix with NMR metabolic fingerprints $y_{i}=\left(y_{i1},y_{i2},\ldots ,y_{ip}\right)$ in its rows, where i=1,…,n indicates the different spectra and $y_{i1}$ to $y_{ip}$ are the corresponding log${}_{2}$-transformed spectral intensities from position 1 to *p*, respectively. In this study, we used the log${}_{2}$-transformed spectra to account for the large dynamic range and heteroscedasticity of the data. Since the logarithmic transformation can not deal with negative values, which occasionally occur in 1D ^1^H NMR spectra due to baseline distortions, we replaced the corresponding numbers by their absolute values. This procedure is not unique, but it only affects regions that do not contain clear metabolite signals. We modeled the data matrix *Y* using a penalized linear regression model:

(1) $$\begin{array}{ccc} \hat{B} & = & \underset{B}{\arg\min}\left\{||Y-{B||}_{F}^{2}+\lambda \sqrt{n}\sum_{j=1}^{p-1}\left|\right|B_{\cdot j}-B_{\cdot j+1}{\left|\right|}_{2}\right\}\; \end{array}$$

where ${\left||.|\right|}_{F}$ is the Frobenius norm and ${\left||.|\right|}_{2}$ is the $l_{2}$ norm, *B* is the parameter matrix that is fitted to the observed data *Y*, $\hat{B}$ is the corresponding estimate, and $B_{\cdot j}$ indicates the *j*th column of *B*. The penalty term $\lambda \sqrt{n}\sum_{j=1}^{p-1}\left|\right|B_{\cdot j}-B_{\cdot j+1}{\left|\right|}_{2}$ [15] was added to segment the data into equal segments across all spectra, which could be understood as follows. Firstly, when we use a limit of n=1, the penalty becomes $\lambda \sum_{j=1}^{p-1}\left|B_{1j}-B_{1,j+1}\right|$, which is a standard fused LASSO regularization, as introduced by [16] and as used for data smoothing or hot spot detection in array comparative genomic hybridization (CGH) data, for example [17]. Secondly, a group penalty term $\sum_{j}\sqrt{\left|\right|\beta_{\left(j\right)}{\left|\right|}_{2}^{2}}=\sum_{j}\sqrt{\beta_{1(j)}^{2}+\ldots +\beta_{n_{j}\left(j\right)}^{2}}$ [18] is used to penalize parameter groups *j*, which consist of $n_{j}$ parameters, and as a consequence, either all of the parameters of a group become zero or they are all determined to be unequal zero. The penalty $\lambda \sqrt{n}\sum_{j=1}^{p-1}\left|\right|B_{\cdot j}-B_{\cdot j+1}{\left|\right|}_{2}=\lambda \sqrt{n}\sum_{j=1}^{p-1}\sqrt{\sum_{i=1}^{n}{\left(B_{ij}-B_{i,j+1}\right)}^{2}}$ combines both aspects, i.e., it enforces the sparseness of the parameter differences between adjacent spectral positions and enforces this sparseness simultaneously across all spectra *i*. Note that the coefficient $\lambda \sqrt{n}$ ensures that λ does not scale with *n*.

### 2.1. Algorithm

The optimization problem that is shown in Equation (1) is a convex optimization problem that can be straightforwardly solved using the alternating direction method of multipliers (ADMM) [19]. This algorithm solves convex optimization problems by breaking them into smaller pieces. For this, we constructed the augmented Lagrangian:

(2) $$\begin{array}{c} L_{\rho }\left(B,G,U\right)=||Y-{B||}_{F}^{2}+\lambda \sqrt{n}\sum_{j=1}^{p-1}\sqrt{\sum_{i=1}^{n}G_{ij}^{2}}\\ +\mathrm{Tr}\left[U^{T}\cdot \left(B\cdot D-G\right)\right]+\frac{\rho }{2}||B\cdot D-{G||}_{F}^{2}\;, \end{array}$$

where:

$$D_{ij}=\{\begin{array}{cc} 1 & for\;i=j\\ -1 & for\;i=j+1\\ 0 & else \end{array}\;,$$

for i=1,…,p and j=1,…,p−1, in which we introduced the auxiliary parameter ρ>0 and the auxiliary parameter matrices *U* and $G\in R^{n\times p-1}$. ADMM then yielded the update scheme:

$$B^{\left(k\right)}=\underset{B}{\arg\min}\;L_{\rho }\left(B,G^{\left(k-1\right)},U^{\left(k-1\right)}\right)\;,$$

$$G^{\left(k\right)}=\underset{G}{\arg\min}\;L_{\rho }\left(B^{\left(k\right)},G,U^{\left(k-1\right)}\right)\;,$$

$$U^{\left(k\right)}=U^{\left(k-1\right)}+\rho \left(B^{\left(k\right)}\cdot D-G^{\left(k\right)}\right)\;,$$

for the parameters at iteration *k*. Here, $B^{\left(k\right)}$ and $G^{\left(k\right)}$ have closed analytical solutions, which can be efficiently computed. The final update scheme then reads:

$$\begin{array}{ccc} B^{\left(k\right)} & = & \left(Y+\frac{\rho }{2}\left(G^{\left(k-1\right)}-U^{\left(k-1\right)}/\rho \right)D^{T}\right){\left(1+\frac{\rho }{2}DD^{T}\right)}^{-1}\;,\\ G_{\cdot j}^{\left(k\right)} & = & R_{\frac{\lambda \sqrt{n}}{\rho }}\;\left({\left(B^{\left(k\right)}\cdot D\right)}_{\cdot j}+\frac{U_{\cdot j}^{\left(k-1\right)}}{\rho }\right),\;\mathrm{for}\;j=1,\ldots ,p-1,\\ U^{\left(k\right)} & = & U^{\left(k-1\right)}+\rho \left(B^{\left(k\right)}\cdot D-G^{\left(k\right)}\right)\;, \end{array}$$

where:

$$R_{t}\left(x\right)={\left(1-\frac{t}{{\left||x|\right|}_{2}}\right)}_{+}\;\;x\;,$$

where $R_{t}\left(x\right)$ is the proximal operator of the group LASSO regularization and $A_{\cdot j}$ indicates the *j*th column of matrix *A*. The step-size was defined as ρ. For initialization, we chose matrices $B^{\left(0\right)},\;G^{\left(0\right)}$, and $U^{\left(0\right)}$ with zeros at all positions. The BF algorithm is available as a python implementation from File S1 in the Supplementary Materials and details about the definition of the hyperparameter λ, as well as our convergence analyses of the BF algorithm, can be found in Sections S5 and S6 in the Supplementary Materials.

### 2.2. Metabolomic Data Acquisition and Processing

#### 2.2.1. Datasets

We assessed the performance of our algorithm using three 1D ^1^H NMR metabolic datasets, all of which were acquired using a 600 MHz Bruker Avance III spectrometer (Bruker BioSpin GmbH, Rheinstetten, Germany) that was equipped with a cryogenic probe head and an automatic cooled sample changer. The first dataset consisted of 1D ^1^H NMR spectra from 106 urine specimens that were collected from patients 24 h after cardiac surgery with cardiopulmonary bypass (CPB) use [1]. Of these 106 patients, 34 were diagnosed with postoperative acute kidney injury (AKI). 400 μL of each urine specimen were mixed with 200 μL of 0.1 mol/L phosphate buffer, pH 7.4, and 50 μL of 29.02 mmol/L 3-trimethylsilyl-2,2,3,3,-tetradeuteropropionate (TSP) dissolved in deuterium oxide as internal standard (Sigma-Aldrich, Taufkirchen, Germany), and the 1D ^1^H NMR spectra were acquired using a 1D nuclear Overhauser enhancement (NOESY) pulse sequence with solvent signal suppression by presaturation during relaxation and mixing time [20]. The NMR spectra are available via the publicly accessible MetaboLights database at https://www.ebi.ac.uk/metabolights/ (accessed on 21 October 2012; accession ID: MTBLS24).

The second dataset comprised 1D ^1^H NMR spectra from 85 EDTA plasma specimens that were collected from patients 24 h cardiac surgery with CPB use, who were a subcohort of the 106 patients mentioned above [2]. Out of these 85 patients, 33 were diagnosed with postoperative AKI. Each EDTA plasma specimen was subjected to 10 kDa cut-off filtration to remove macromolecules and subsequent sample preparation, as well as NMR spectral data acquisition, which was performed as described above.

The third dataset consisted of 1D ^1^H NMR spectra from 223 EDTA plasma specimens that were collected at the baseline time point of the German Chronic Kidney Disease (GCKD) study [3,21]. For this dataset, 400 μL of each unfiltered EDTA plasma specimen were mixed with 200 μL of 0.1 mol/L phosphate buffer, pH 7.4, 50 μL of 0.75% (*w/v*) TSP that was dissolved in deuterium oxide, and 10 μL of 81.97 mmol/L formic acid (Sigma-Aldrich, Taufkirchen, Germany), which served as the internal standard for referencing and quantification. The 1D ^1^H NMR spectra were acquired using a Carr–Purcell–Meiboom–Gill (CPMG) pulse sequence to suppress unspecific macromolecular signals. The absolute concentrations of 25 unique metabolites were quantified from these 1D ^1^H NMR spectra, according to the method in [22] and using the Chenomx software suite (Chenomx Inc., Edmonton, AB, Canada). The NMR signals were identified through comparison to reference spectra from pure compounds, which were available from the Chenomx software suite. The NMR spectra are available from the MetaboLights database at https://www.ebi.ac.uk/metabolights/ (accessed on 26 June 2019; accession ID: MTBLS798).

#### 2.2.2. Feature Extraction

The initial feature extraction for all three datasets was performed via equidistant binning (with a bin width of 0.001 ppm) using Amix 3.9.13 (Bruker BioSpin GmbH, Rheinstetten, Germany), followed by data import into *R* version 3.6.0, https://cran.r-project.org [23] (accessed on 27 July 2022).

The spectral region from 9.5 to −0.5 ppm of the AKI urine 1D ^1^H NMR dataset was split into 9999 even bins. The spectral regions from 6.5 to 4.5 ppm, which corresponded to the remaining water and broad urea signals, and the TSP region from 0.5 to −0.5 ppm were excluded, which resulted in a total of 7000 bins. A probabilistic quotient normalization (PQN) [24] was applied to reduce sample-to-sample variations that were caused by differences in fluid intake.

For the AKI plasma 1D ^1^H NMR dataset, the spectral region from 9.5 to −0.5 ppm was split into 9999 even bins (the raw bucket table has been published in [25]). The spectral intensities were normalized to the internal standard TSP to correct for variations in spectrometer performance [2]. The spectral region from 6.2 to 4.6 ppm, which corresponded to the remaining water and broad urea signals, and the TSP region from 0.5 to −0.5 ppm were excluded, which resulted in a total of 7400 bins. After the application of the different binning methods, the regions of 3.82–3.76 ppm, 3.68–3.52 ppm, 3.23–3.20 ppm, and 0.75–0.72 ppm, which corresponded to filter residues and free EDTA, were excluded prior to further statistical analysis.

The GCKD 1D ^1^H NMR spectra were referenced and normalized to the internal standard formic acid to correct for variations in spectrometer performance [3,21]. The spectral region from 9.5 to 0.5 ppm was evenly split into 9000 bins and the spectral region from 6.0 to 4.5 ppm, which corresponded to the remaining water and broad urea signals, was excluded, which resulted in a total of 7499 bins.

Each of the three datasets was subjected to seven different binning approaches to yield the following datasets: three different BF datasets that corresponded to the three penalization parameters λ=1, λ=2.5, and λ=5; two different equidistant binning datasets with bucket widths of 0.01 ppm and 0.02 ppm, which were created by summing the individual spectral intensity values of 10 or 20 adjacent buckets with 0.001 ppm widths into one bucket, respectively; one SRV dataset employing the *R* package *mQTL* [26] with rectangular binning, which corresponded to the default settings; one JBA dataset employing the *R* package *MWASTools* [27]. To ensure method comparability, the minimum bucket size was set to a width of 0.003 ppm (i.e., three bins with 0.001 ppm widths) and the spectral intensity values were summed using the SRV, JBA, and BF algorithms.

## 3. Results

### 3.1. Bucket Fuser Dynamically Constructs NMR Metabolite Features

In this first subsection, we discuss the output that was generated by BF. BF relies on a single positive parameter, λ, which is the regularization parameter of the penalized linear regression problem in Equation (1). Note that for λ=0, BF returns the input matrix *Y* since every entry of $Y_{ij}$ is described by an individual parameter $B_{ij}$, i.e., the residual sum of squares $||Y-{B||}_{F}^{2}$ becomes zero. When we increase the value of λ, the fit parameters $B_{ij}$ become increasingly penalized by the regularization term $\lambda \sqrt{n}\sum_{j=1}^{p-1}\sqrt{\sum_{i=1}^{n}{\left(B_{ij}-B_{i,j+1}\right)}^{2}}$. First, we applied BF to the urinary AKI data (dataset 1) and produced results for three ad-hoc λ values (λ=1, λ=2.5, and λ=5) for demonstration purposes.

**Figure 1:**
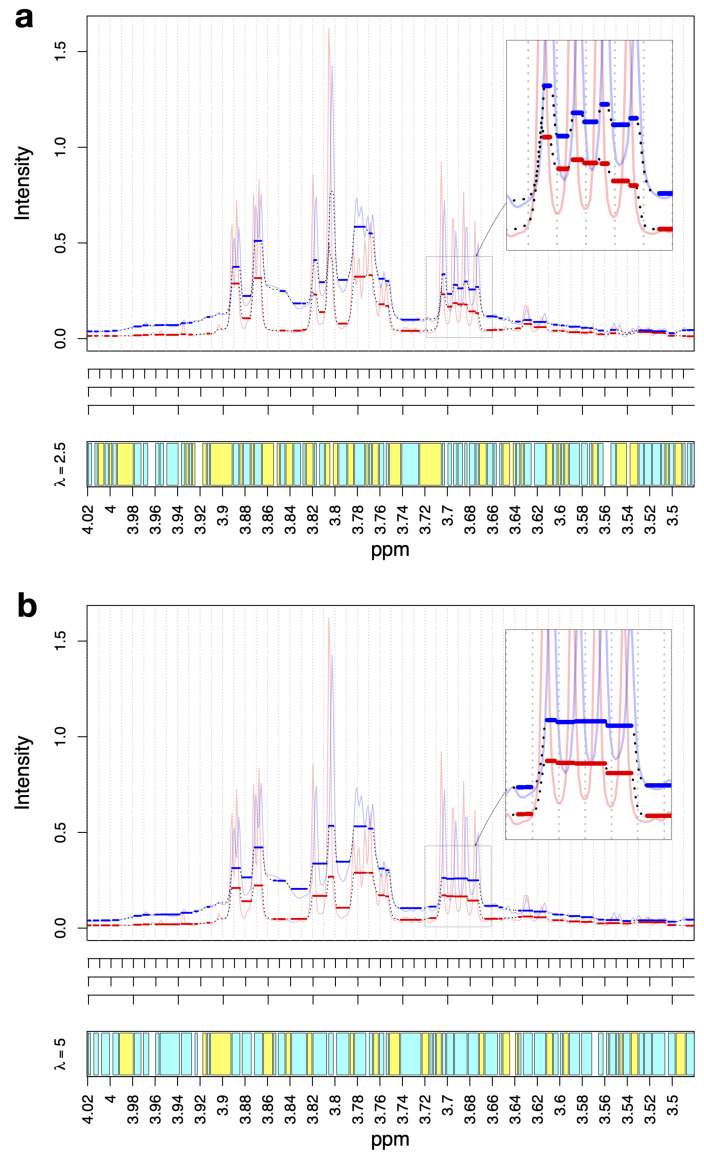
(**a**,**b**) Exemplary NMR spectral regions that ranged from 4 ppm to 3.5 ppm, together with their corresponding BF fits for λ=2.5 and λ=5, respectively. The thin blue and red lines show the spectral intensities of two exemplary NMR spectra from the urinary AKI dataset. The black dotted lines show the corresponding BF fits, along which the plateaus of the fits are additionally highlighted as thick blue and red lines. The ticks in the middle of the figures correspond to the standard equidistant binning with bin sizes of 0.01 ppm, 0.02 ppm, and 0.04 ppm (from top to bottom, respectively). The lower parts of the figures display the detected consensus plateaus in cyan, which start and end at the same positions for all included spectra. The yellow blocks represent the regions that did not correspond to plateaus but were also retained for subsequent analysis. For both the yellow and cyan regions, we neglected those with widths ≤0.002 ppm, which are plotted as white blocks. The inserted figures show these dotted regions in detail.

Figure 1a,b show the spectral region that ranged from 4.0 to 3.5 ppm for two exemplary AKI urine spectra for parameters λ=2.5 and λ=5, respectively. The corresponding fit for λ=1 is shown in Figure S1 in the Supplementary Materials. This region covered, for example, signals from D-mannitol, which was administered to all patients during cardiac surgery, and myo-inositol. In all three figures, the fitted spectra are shown as black dotted lines that follow the observed spectra, which are shown as thin blue and red lines. From these figures, we could observe three general properties of BF:

(1) BF fits plateaus, as shown by the thick blue and red lines;
(2) The plateaus start and end at the same position for all spectra;
(3) The regularization parameter λ calibrates the plateau width: λ=5 yields larger plateaus than λ=2.5 and λ=2.5 yields larger plateaus than λ=1.

The plateaus are consensus regions that define the variables for the subsequent data analysis. The regions in which no plateaus are built can be either neglected from further analysis or included as additional spectral features. The bottom panels of Figure 1a,b and S1 show the consensus regions in cyan and the non-consensus regions in yellow. For the analyses that are presented in this article, we retained both the cyan and yellow regions, i.e., we retained both the plateau regions and non-plateau regions. Of these regions, we neglected those with widths ≤0.002 ppm, which are plotted as white blocks, to be consistent with the minimal bin width that was used in SRV and JBA. Note that λ→∞ builds a single plateau that covers the whole spectrum, i.e., one single consensus region is returned. In contrast, λ=0 does not build any plateaus and again, a single consensus region is returned.

### 3.2. Bucket Fuser Improved Signal Extraction

In this subsection, we present our comparison of the extracted NMR metabolite features and the absolutely quantified metabolite concentrations in plasma samples from 223 patients who were included in the German Chronic Kidney Disease (GCKD) study. In total, quantified values were available for 25 metabolites.

We compared seven different methods for signal extraction: BF using the penalty parameters λ=1, λ=2.5, and λ=5; JBA, which is one of the most recent adaptive binning approaches [14]; SRV, which is one of the most frequently used adaptive binning methods; two standard equidistant binning methods with bin sizes of 0.01 ppm and 0.02 ppm. Table 1 lists the number of metabolite features that were extracted using each of these methods. The number of extracted features ranged from 375 (equidistant binning with a 0.02 ppm bin size) to 808 (BF with λ=5). Note that using a higher penalty parameter λ for BF did not necessarily return fewer metabolite features. However, the average width of the plateau regions grew with λ: for BF with λ=1, we obtained an average plateau width of 0.0038 ppm; for λ=2.5, we obtained an average plateau width of 0.0057 ppm; for λ=5, we obtained an average plateau width of 0.0064 ppm. We further observed that the plateau widths remained stable with respect to the number of samples *n* for λ=1 (dotted line), λ=2.5 (dashed line), and λ=5 (solid line), as shown in Figure S2 in the Supplementary Materials.

Next, we tested the associations between the metabolite features and the absolute metabolite concentrations from the complete GCKD dataset. For this purpose, we selected, for each quantified metabolite, the binned metabolite feature which correlates best, where we used Spearman’s correlation to properly deal with outlier values. The correlations for all seven methods are listed in Table 2.

For several metabolites, the performance of the binning approaches deviated substantially, including pyruvate (for which the correlations ranged from r=0.692 (equidistant binning with a bin width of 0.02 ppm) to r=0.968 (BF with λ=2.5)) and betaine (for which the correlations ranged from r=0.221 (equidistant binning with a bin width of 0.02 ppm) to r=0.689 (JBA)). Generally, there was not a single extraction method that consistently worked best for all quantified metabolites. However, by comparing all seven methods, we observed that BF (λ=1) extracted metabolite features with the highest correlations for 7 of the 25 quantified metabolites. BF (λ=2.5) returned the highest correlations for eight metabolites, BF (λ=5) returned the highest correlations for five metabolites, JBA returned the highest correlations for five metabolites, SRV returned the highest correlations for a single metabolite, equidistant binning with a 0.01 ppm bin size returned the highest correlations for one metabolite, and equidistant binning with a 0.02 ppm bin size did not return the highest correlations for any metabolite. For 3-hydroxybutyrate and acetoacetate, BF with λ=1 and JBA performed the best and thus, we highlighted these two methods. Next, we repeated this analysis but instead individually compared BF with different regularization parameters to the other selected methods. These results are summarized in Table 3 and show that the BF approaches delivered superior performance for all selected regularization parameters, followed by JBA, SRV, and the equidistant binning method. The corresponding scatter plots for each of the quantified metabolites using each of the seven binning methods are shown in Figures S3–S9 in the Supplementary Materials, respectively.

### 3.3. Metabolite Identification

We further evaluated the identities of the metabolite features with the highest correlations to the absolutely quantified metabolite concentrations, which are presented in Table S1 in the Supplementary Materials. The signals of some metabolites (e.g., trimethylamine N-oxide, asparagine, and threonine) could not be correctly extracted by any of the selected methods. This issue occurred when the metabolite signals under consideration appeared in crowded regions of the spectrum or overlapped completely with other signals. Small molecules with chemically equivalent protons that resulted in only one singlet were especially susceptible to this effect, such as trimethylamine N-oxide. This gave rise to a singlet signal at approximately 3.27 ppm, which was overlaid by a singlet of betaine and a glucose multiplet (Figure S10 in the Supplementary Materials). Absolute quantification circumvented this problem by deconvoluting overlapping signals; however, the metabolite feature extraction methods did not employ spectral deconvolution and thus, only yielded rather small correlation coefficients for trimethylamine N-oxide, even when the best associated metabolite feature was trimethylamine N-oxide (i.e., when using BF (λ=1), BF (λ=2.5), BF (λ=5), and JBA). On the other hand, the signals of other metabolites (such as glucose, lactate, and tyrosine) were extracted with high Spearman’s correlations by all selected methods. These metabolites often give rise to multiple signals with characteristic chemical shifts and hardly any overlap with signals of other metabolites. Considering all 25 metabolites that were investigated in this study, BF (λ=2.5) correctly identified the largest number with 21 metabolites, followed by BF (λ=1) and BF (λ=5) with 19 each, and equidistant binning with a bin width of 0.01 ppm with 18 correctly identified metabolites. The lowest number of correct assignments was accomplished by equidistant binning with a bin width of 0.02 ppm as it only identified 13 metabolites correctly.

### 3.4. The Bucket Fuser Can Deal with Small Sample Sizes

The equidistant binning method cuts the spectrum into regions of equal width and thus, defines the same regions regardless of the provided spectra. In contrast, BF, JBA, and SRV construct metabolic features from the data; thus, they extract spectral regions that inherently depend on the provided spectra. In this part of the study, we repeated the comparison to the absolutely quantified metabolite concentrations for 25 metabolites, as shown previously, but systematically reduced the number of training samples in a subsampling approach. Thus, we investigated how both the choice of training samples and the number of training samples influenced feature extraction.

We performed the following experiment. We repeatedly drew n∈{5,10,20,40,80,160} from the 223 plasma samples from the GCKD cohort and used them to train a spectral binning algorithm. These binning methods were then applied to the remaining samples to evaluate the binning performance. This was validated by comparing the results to the absolutely quantified metabolite concentrations using Spearman’s correlation, as before. Figure S11 in the Supplementary Materials summarizes the binning performance for each of the 25 quantified metabolites as a function of the number of training samples *n* using BF with λ=1 (dotted blue lines), λ=2.5 (dashed blue lines), λ=5 (solid blue lines), and JBA (solid red lines). The corresponding results for SRV (solid purple lines) and the equidistant binning method with bin sizes of 0.01 ppm (green solid lines) and 0.02 ppm (green dashed lines) are shown in Figure S12 in the Supplementary Materials. The dots represent the median values that were obtained from 50 subsampling runs and the whiskers correspond to the upper and lower quartiles.

We observed that the equidistant binning method, JBA, and SRV were barely affected by the number of training samples. In fact, JBA even showed a better performance with smaller sample sizes, which was evident by the *n* versus *r* plots for creatinine and glutamine, for example. The BF approaches were affected by sample size for some of the 25 metabolites. Metabolites that showed a stronger sample-size dependence included creatinine, dimethylamine, and histidine. However, for all three λ values, BF still performed better than JBA, even with sample sizes as small as n=5. The strongest dependence on sample size was observed for histidine, for which the binning performance increased substantially with training sample size.

In summary, we observed that BF depended more on sample size than the other methods that were investigated in this study; however, even for very small sample sizes, BF still outperformed the other methods for the majority of the metabolites.

### 3.5. The Bucket Fuser Improved the Detection of Metabolic Biomarkers for Acute Kidney Injury after Cardiac Surgery

**Figure 2:**
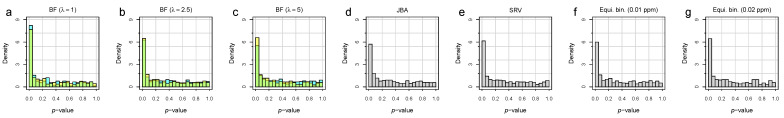
The *p*-value distributions of AKI versus non-AKI patients after cardiac surgery in urine specimens: (**a**–**g**) the different binning approaches (BF with λ=1, BF with λ=2.5, BF with λ=5, JBA, SRV, equidistant binning with a bin size of 0.01 ppm, and equidistant binning with a bin size of 0.02 ppm, respectively). For the BF method, the same color-coding was applied as that in Figure 1, for example. Note that the light green bars correspond to the overlapping regions of the cyan and yellow bars.

**Figure 3:**
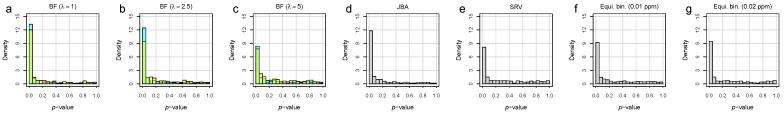
The *p*-value distributions of AKI versus non-AKI patients after cardiac surgery in plasma specimens: (**a**–**g**) the different binning approaches (BF with λ=1, BF with λ=2.5, BF with λ=5, JBA, SRV, equidistant binning with a bin size of 0.01 ppm, and equidistant binning with a bin size of 0.02 ppm, respectively). For the BF method, the same color-coding was applied as that in Figure 1, for example. Note that the light green bars correspond to the overlapping regions of the cyan and yellow bars.

In the next step, we assessed the performance of different feature extraction methods using differential metabolite analysis. Differences in either the urine or plasma metabolomes of adults who had undergone cardiac surgery with CPB use, who did or did not experience a subsequent acute kidney injury (AKI) event, were investigated using a two-sample unequal variance *t*-test. Note that binning methods are designed to extract meaningful metabolic variables and as such, are expected to increase the number of metabolite variables that contain true biological signals compared to variables that just contain noise. Consequently, we expected that the *p*-value distributions at low *p*-values would peak stronger for better binning performances. Figure 2 and Figure 3 depict the distributions of the raw *p*-values for the urinary and plasma AKI datasets, respectively, using the seven different feature binning approaches. For BF, we show the *p*-value distributions for plateau (cyan) and non-plateau (yellow) regions separately. For each investigated method, the numbers of metabolite features that were extracted from the two datasets are presented in Table S2 in the Supplementary Materials.

For both datasets, the BF method with λ=1 obtained the *p*-value distribution with the highest density for raw *p*-values between 0 and 0.05, taking into account the predominantly uniform raw *p*-value distribution between 0.05 and 1. This effect was even more pronounced for the plateau regions (cyan). This result was followed by BF with λ=2.5 and equidistant binning with a 0.02 ppm bucket width for the urinary AKI dataset and by BF with λ=2.5 and JBA for the plasma AKI dataset. BF λ=5 was inferior compared to BF with λ=1 and λ=2.5 and delivered a performance that was similar to that of SRV and the equidistant binning method. The uniform raw *p*-value distributions were obtained for all binning methods following random class label permutation, as depicted in Figures S13 and S14 in the Supplementary Materials.

Finally, we analyzed the performance of the different binning approaches in the context of multivariate data analysis (MVA), namely the classification of “yes” versus “no” for AKI after cardiac surgery with CPB use. It is worth mentioning that it is not clear a priori whether superior binning performance also results in improved classification performance as classification performance can be determined by small sets of bins and their extraction from data. However, as shown in this subsection, BF can be used to systematically improve prediction performance for MVA.

**Figure 4:**
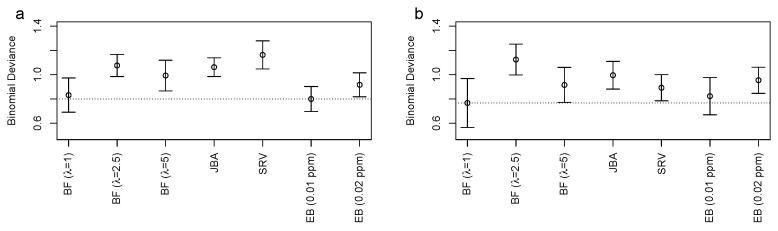
The binomial deviances (*y*-axis) from the leave-one-out cross-validation using the different binning approaches (*x*-axis) for the plasma (**a**) and urinary (**b**) AKI data. The dotted horizontal lines correspond to the best method for the plasma (EB (0.01 ppm)) and urine (BF (λ=1)) data. The error bars correspond to ±1 standard deviation.

**Figure 5:**
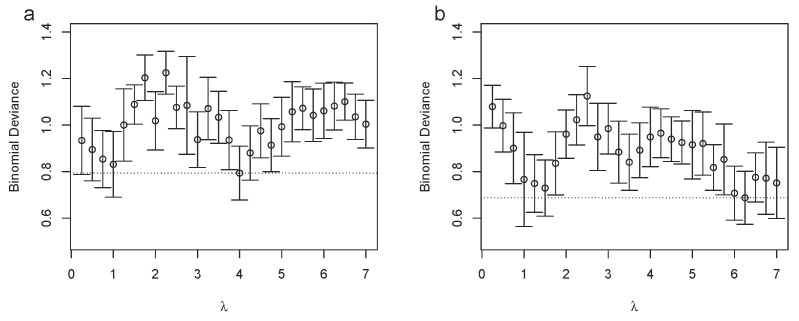
The binomial deviances (*y*-axis) from the leave-one-out cross-validation using BF with regularization parameter (λ) values between 0.25 and 7.0 (*x*-axis) for the plasma (**a**) and urinary (**b**) AKI data. The dotted horizontal line corresponds to the lowest observed binomial deviance across all λ values. The error bars correspond to ±1 standard deviation.

We applied binomial zero-sum LASSO regression, which is a multivariate normalization invariant classification approach that is based on the binomial LASSO regression augmented by an additional constraint on the regression parameters, i.e., the so-called zero-sum constraint [28,29]. This approach turned out to be particularly suited for NMR spectroscopic data since it could compensate for the systematic errors from sample dilution and normalization [25]. We used the implementation that was available in the *R* package zeroSum from https://github.com/rehbergT/zeroSum (accessed on 15 August 2022), using the standard parameters. We directly extracted the minimum leave-one-out cross-validation error (binomial deviance) for the different binning methods and the results are shown in Figure 4a,b for the plasma and urinary AKI data, respectively (see Figures S15a–g and S16a–g in the Supplementary Materials for the corresponding hyperparameter calibration). We observed that for the plasma data, the EB (0.01 ppm) performed best with a binomial deviance of 0.80±0.10, followed closely by Bucket Fuser (λ=1) with a binomial deviance of 0.83±0.14. For the urinary AKI data, BF (λ=1) performed best with 0.77±0.20, closely followed by EB (0.01 ppm) with 0.82±0.15. The remaining methods performed worse, with binomial deviances between 0.92±0.10 (EB (0.02 ppm)) and 1.16±0.12 (SRV) for the plasma data and between 0.89±0.11 (SRV) and 1.12±0.13 (BF (λ=2.5)) for the urine data. Thus, for MVA, we did not observe a single best-performing binning strategy and the performance depended on the parameter choice, i.e., λ for BF and bin size for the equidistant binning method. To substantiate this finding, we systematically analyzed how classification performance depended on the choice of λ and the results are shown in Figure 5a,b for the plasma and urinary AKI data, respectively, from which we extracted the minimum binomial deviances using internal leave-one-out cross-validation for λ∈{0.25,0.5,…,7}. We observed that the performance could be improved for both datasets, with a minimum binomial deviance of 0.79±0.12 using λ=4 for the plasma dataset and 0.69±0.11 using λ=6.25 for the urinary dataset.

## 4. Discussion and Conclusions

We developed Bucket Fuser as an alternative method for extracting metabolic features from 1D ^1^H NMR metabolic data. We compared the developed method to other state-of-the-art approaches and demonstrated its superior performance using absolutely quantified metabolite concentrations. Moreover, we studied two realistic applications in which metabolite concentrations in urine and blood from individuals who suffered an AKI event after cardiac surgery were compared to the metabolite concentrations in samples from patients who did not develop AKI. From this comparison, the *p*-value distributions also indicated the superior performance of BF compared to the other state-of-the-art methods. In this context, it was interesting to observe that the *p*-value distributions of the plateau regions showed the strongest peaks. The peaks were less pronounced for non-plateau regions. This finding was not surprising since BF predominantly built consensus plateau regions around peaks that occurred at the same position across multiple spectra. Thus, a plateau that was determined by BF also provided additional evidence of consistent signals within this region across multiple spectra. BF depends on a single parameter, i.e., the regularization parameter λ. Similarly, the equidistant binning method depends on bin size and JBA and SRV depend on correlation thresholds, which are both chosen by the user. To improve the usability of BF, the regularization parameter was defined so that it yielded stable plateau widths for different sample sizes. Consequently, the regularization parameter might not need to be recalibrated for new datasets. In practice, we observed that values around the order of ∼1 performed reasonably well for an initial bin width of 0.001 ppm.

BF was implemented using a highly efficient algorithm that is based on the alternating direction method of multipliers (ADMM). For smaller datasets and reasonable regularization parameters (∼100 samples and λ=1–5), BF could be run within minutes on ordinary desktop computers. However, it is based on a high-dimensional regression setup with p×n parameters and complex group LASSO regularization terms. Other methods, such as JBA and SRV, are usually computationally more efficient and the computation time restricts the hyperparameter tuning of BF, i.e., hyperparameter tuning is only an option for expert users with optimized computing resources. The BF procedure in its current implementation could be further optimized computationally and does not entirely exploit state-of-the-art computing resources, such as graphical processing units. Moreover, there have been algorithmic advances, such as accelerated alternating direction method of multipliers (A2DM2) [30]. The integration of both of these improvements in future research could substantially increase the computation speed. We further emphasize that the application of the BF algorithm is not restricted to the analysis of 1D ^1^H NMR spectroscopic data as there are numerous data types that require segmentation steps for analyses (e.g., array CGH data). BF could be straightforwardly applied to such data and offers the additional benefit that data segmentation would be chosen consistently across all measurements, which could be particularly valuable for machine learning applications. Moreover, BF could be modified to account for 2D data, which could be a promising future direction of research, e.g., for the analysis of 2D NMR spectroscopic data.

It is worth mentioning that Bucket Fuser, as well as all of the other methods that were considered in this study, works in the frequency domain of NMR spectra. Alternative approaches to NMR feature extraction have been developed in the time domain, e.g., the complete reduction to amplitude frequency table (CRAFT) approach [31]. A thorough benchmarking of both frequency- and time-domain methods warrants future work. Likewise, the combination of Bucket Fuser and a time-domain feature extraction method, such as CRAFT, was beyond the scope of this manuscript but could enhance the performance of both methods.

In summary, BF is a new and promising approach for the extraction of metabolic features from NMR metabolic data. It could improve the resolution of metabolic features and has the potential to extract new metabolic features that are not captured by current state-of-the-art methods.

## Figures and Tables

**Table 1:** The number of metabolite features that were extracted from the 1D ^1^H NMR spectra of 223 plasma samples from the GCKD cohort using different binning approaches. The BF results are presented in the form “number of plateau regions + number non-plateau regions = number of features”.

BF (λ = 1)	BF (λ = 2.5)	BF (λ = 5)	SRV	JBA	EB (0.01 ppm)	EB (0.02 ppm)
360 + 261 = 621	398 + 234 = 632	507 + 301 = 808	531	538	749	375

**Table 2:** The Spearman’s correlations to the absolutely quantified metabolite concentrations using BF with λ=1, BF with λ=2.5, BF with λ=5, JBA, SRV, and the equidistant binning method with bin sizes of 0.01 ppm and 0.02 ppm. The highest correlations for each of the 25 metabolites are highlighted in bold.

	BF (λ = 1)	BF (λ = 2.5)	BF (λ = 5)	JBA	SRV	EB (0.01 ppm)	EB (0.02 ppm)
3-Hydroxybutyrate	**0.768**	0.720	0.689	**0.768**	0.600	0.547	0.498
Acetate	0.757	**0.983**	0.968	0.966	0.908	0.946	0.892
Acetoacetate	**0.670**	0.664	0.610	**0.670**	0.611	0.614	0.603
Acetone	0.528	**0.748**	0.568	0.530	0.472	0.455	0.350
Alanine	0.680	0.927	**0.947**	0.722	0.915	0.926	0.905
Asparagine	0.685	0.662	0.635	0.563	**0.698**	0.683	0.659
Betaine	0.509	0.637	0.480	**0.689**	0.481	0.630	0.221
Carnitine	0.678	0.419	0.427	**0.692**	0.412	0.444	0.445
Creatine	**0.929**	0.907	0.584	0.909	0.842	0.763	0.626
Creatinine	0.772	**0.893**	0.881	0.443	0.749	0.703	0.619
Dimethylamine	**0.895**	0.649	0.649	0.598	0.684	0.587	0.592
Glucose	**0.990**	0.990	0.989	0.987	0.989	0.990	0.988
Glutamine	0.873	0.870	**0.905**	0.612	0.779	0.897	0.878
Glycine	**0.838**	0.808	0.741	0.332	0.778	0.655	0.543
Histidine	0.548	**0.764**	0.523	0.453	0.571	0.558	0.631
Isobutyrate	**0.845**	0.793	0.568	0.445	0.687	0.618	0.522
Isoleucine	0.866	**0.911**	0.808	0.735	0.762	0.792	0.790
Lactate	0.988	0.989	0.989	0.979	0.983	**0.990**	0.985
Phenylalanine	0.850	0.874	**0.888**	0.819	0.818	0.810	0.800
Proline	0.754	**0.937**	0.699	0.676	0.871	0.680	0.609
Pyruvate	0.884	**0.968**	0.941	0.884	0.931	0.857	0.692
Threonine	0.494	**0.502**	0.490	0.426	0.472	0.490	0.474
TMAO	0.282	0.401	0.403	**0.449**	0.279	0.400	0.231
Tyrosine	0.931	0.941	**0.947**	0.816	0.935	0.939	0.924
Valine	0.811	0.947	**0.961**	0.725	0.924	0.952	0.952

**Table 3:** The three different BF approaches (with λ=1, λ=2.5, and λ=5) individually compared to JBA, SRV, and the equidistant binning method. The numbers indicate how often each method was selected as the “best performing”.

	BF (λ = 1)	BF (λ = 2.5)	BF (λ = 5)	JBA	SRV	EB (0.01 ppm)	EB (0.02 ppm)
BF (λ=1)	11	-	-	7	3	5	1
BF (λ=2.5)	-	14	-	6	2	3	0
BF (λ=5)	-	-	11	6	5	2	1

## Data Availability

The NMR spectra of the AKI urine dataset are available from the publicly accessible MetaboLights database at https://www.ebi.ac.uk/metabolights/ (accessed on 21 October 2012; accession ID: MTBLS24). The NMR spectra of the GCKD dataset are available from the MetaboLights database at https://www.ebi.ac.uk/metabolights/ (accessed on 26 June 2019; accession ID: MTBLS798). The raw bucket table of the AKI plasma NMR dataset was published in [25].

## Supplementary Materials

The following are available online at https://www.mdpi.com/article/10.3390/metabo12090812/s1, Section S1: The current GCKD investigators and collaborators who are involved in the GCKD Study, Section S2: The Python implementation of the Bucket Fuser algorithm, Section S3: Supplementary Tables, Table S1: The Spearman’s correlations to the absolutely quantified metabolite concentrations using BF with λ=1, BF with λ=2.5, BF with λ=5, JBA, SRV, and the equidistant binning method with the bin sizes of 0.01 ppm and 0.02 ppm, Table S2: The number of metabolite features that were extracted from the 1D ^1^H NMR spectra of the (a) urinary and (b) plasma AKI datasets using the different binning approaches, Section S4: Supplementary Figures, Figure S1: The exemplary NMR spectral regions that ranged from 4 ppm to 3.5 ppm, together with their corresponding BF fits for λ=1, Figure S2: The average plateau widths versus the number of training samples for λ=1, λ=2.5, and λ=5 using the GCKD dataset, Figure S3: The comparison of the integrals of the spectral features that were constructed by Bucket Fuser using λ=1 and the absolutely quantified metabolite concentrations for the 25 metabolites, Figure S4: The comparison of the integrals of the spectral features that were constructed by Bucket Fuser using λ=2.5 and the absolutely quantified metabolite concentrations for the 25 metabolites, Figure S5: The comparison of the integrals of the spectral features that were constructed by Bucket Fuser using λ=5 and the absolutely quantified metabolite concentrations for the 25 metabolites, Figure S6: The comparison of the integrals of the spectral features that were constructed by JBA and the absolutely quantified metabolite concentrations for the 25 metabolites, Figure S7: The comparison of the integrals of the spectral features that were constructed by SRV and the absolutely quantified metabolite concentrations for the 25 metabolites, Figure S8: The comparison of the integrals of the spectral features that were constructed by the equidistant binning method (bin size = 0.01 ppm) and the absolutely quantified metabolite concentrations for the 25 metabolites, Figure S9: The comparison of the integrals of the spectral features that were constructed by the equidistant binning method (bin size = 0.02 ppm) and the absolutely quantified metabolite concentrations for the 25 metabolites, Figure S10: The exemplary NMR spectral region that ranged from 3.29 to 3.25 ppm, comprising the metabolite signals of D-glucose, betaine, trimethylamine-N-oxide, and myo-inositol, Figure S11: The Spearman’s correlations between the absolutely quantified metabolite concentrations and the integrals of the corresponding spectral features that were constructed by the different binning approaches, according to the number of training samples *n* for the 25 absolutely quantified metabolites from the GCKD dataset, Figure S12: The Spearman’s correlations between the absolutely quantified metabolite concentrations and the integrals of the corresponding spectral features that were constructed by the different binning approaches, according to the number of training samples *n* for the 25 absolutely quantified metabolites from the GCKD dataset, Figure S13: The *p*-value distributions of AKI versus non-AKI after cardiac surgery, based on the permuted urine data, Figure S14: The *p*-value distributions of AKI versus non-AKI after cardiac surgery, based on permuted plasma data, Figure S15: The hyperparameter calibration for the plasma AKI dataset, Figure S16: The hyperparameter calibration for the urinary AKI dataset. Section S5: The relationship between the choice of logarithmic base and the regularization parameter λ, Section S6: The convergence analysis of the BF algorithm.
