# Supplementary material for: Bucket Fuser: Statistical Signal Extraction for 1D 1H NMR Metabolomic Data
Source: Metabolites. 2022 Aug 29;12(9):812. doi: 10.3390/metabo12090812 (PMC9501206; doi:10.3390/metabo12090812)
Supplement: Supplementary file 1 [file metabolites-12-00812-s001.zip › supplement.pdf]

Supplementary Information for "Bucket Fuser: statistical signal extraction for 1D ^{1}H NMR metabolomics data"

1 Current GCKD Investigators and Collaborators with the GCKD Study

University of Erlangen: Kai-Uwe Eckardt, Heike Meiselbach, Markus P. Schneider, Mario Schiffer, Hans-Ulrich Prokosch, Barbara Bärthlein, Andreas Beck, André Reis, Arif B. Ekici, Susanne Becker, Dinah Becker-Grosspitsch, Ulrike Alberth-Schmidt, Birgit Hausknecht, Anke Weigel;
University of Freiburg: Gerd Walz, Anna Köttgen, Ulla T. Schultheiß, Fruzsina Kotsis, Simone Meder, Erna Mitsch, Ursula Reinhard;
RWTH Aachen University: Jürgen Floege, Turgay Saritas;
Charité, University Medicine Berlin: Elke Schaeffner, Seema Baid-Agrawal, Kerstin Theisen;
Hannover Medical School: Hermann Haller, Jan Menne;
University of Heidelberg: Martin Zeier, Claudia Sommerer, Johanna Theilinger;
University of Jena: Gunter Wolf, Martin Busch, Rainer Paul;
Ludwig-Maximilians University of München: Thomas Sitter;
University of Würzburg: Christoph Wanner, Vera Krane, Antje Börner-Klein, Britta Bauer;
Medical University of Innsbruck, Division of Genetic Epidemiology: Florian Kronenberg, Julia Raschenberger, Barbara Kollerits, Lukas Forer, Sebastian Schönherr, Hansi Weissensteiner;
University of Regensburg, Institute of Functional Genomics: Peter Oefner, Wolfram Gronwald;
Institute of Medical Biometry, Informatics and Epidemiology, Medical Faculty, University of Bonn: Matthias Schmid, Jennifer Nadal.

2 The Python implementation of the Bucket Fuser algorithm

The Python implementation of the Bucket Fuser algorithm and a comprehensive tutorial are available in supplementary file S1.

3 Supplementary Tables

Table S1: Spearman's correlations to absolutely quantified metabolite concentrations for BF with $\lambda = 1$, BF with $\lambda = 2.5$, BF with $\lambda = 5$, JBA, SRV, and equidistant binnings with bin sizes 0.01 ppm and 0.02 ppm. Here, 3-HB = 3-hydroxybutyrate. Further provided are the spectral positions of the bins with largest absolute Spearman's correlation coefficients and the corresponding metabolite identities.

	BF ($\lambda = 1$)			BF ($\lambda = 2.5$)			BF ($\lambda = 5$)		
	correlation coef.	spectral position	metabolite	correlation coef.	spectral position	metabolite	correlation coef.	spectral position	metabolite
3-HB	0.768	ppm_2.3974-ppm_2.3954	3-HB, carnitine, glutamate	0.720	ppm_4.1694-ppm_4.1664	3-HB, lactate, proline	0.689	ppm_4.1564-ppm_4.1544	3-HB, lactate, proline
Acetate	0.757	ppm_1.9244-ppm_1.9194	acetate	0.983	ppm_1.9274-ppm_1.9244	acetate	0.968	ppm_1.9274-ppm_1.9224	acetate
Acetoacetate	0.670	ppm_2.3814-ppm_2.3794	3-hydroxyisovalerate, pyruvate, succinate	0.664	ppm_2.3794-ppm_2.3774	3-hydroxyisovalerate, pyruvate, succinate	0.610	ppm_2.3794-ppm_2.3744	3-hydroxyisovalerate, pyruvate, succinate
Acetone	0.528	ppm_2.3314-ppm_2.3294	2-hydroxyglutarate, glutamate, proline	0.748	ppm_2.2354-ppm_2.2334	acetone, macromolecules	0.568	ppm_2.2374-ppm_2.2314	acetone, macromolecules
Alanine	0.680	ppm_1.4874-ppm_1.4844	alanine	0.927	ppm_1.4934-ppm_1.4914	alanine	0.947	ppm_1.4814-ppm_1.4784	alanine
Asparagine	0.685	ppm_2.1444-ppm_2.1424	glutamate, glutamine	0.662	ppm_2.1444-ppm_2.1384	glutamate, glutamine	0.635	ppm_2.1514-ppm_2.1284	glutamate, glutamine
Betaine	0.509	ppm_3.2814-ppm_3.2694	glucose, betaine, myo-inositol, trimethylamine-N-oxide	0.637	ppm_3.2784-ppm_3.2704	glucose, betaine, myo-inositol, trimethylamine-N-oxide	0.480	ppm_3.2784-ppm_3.2724	glucose, betaine, myo-inositol, trimethylamine-N-oxide
Carnitine	0.678	ppm_2.4484-ppm_2.4464	glutamine, carnitine	0.419	ppm_3.2014-ppm_3.1994	macromolecule (?)	0.427	ppm_3.0504-ppm_3.0434	creatinine, creatine
Creatine	0.929	ppm_3.9394-ppm_3.9374	creatine	0.907	ppm_3.9404-ppm_3.9374	creatine	0.584	ppm_3.0504-ppm_3.0434	creatinine, creatine
Creatinine	0.772	ppm_3.0494-ppm_3.0474	creatinine	0.893	ppm_4.0624-ppm_4.0594	creatinine	0.881	ppm_4.0634-ppm_4.0584	creatinine
Dimethylamine	0.895	ppm_2.7304-ppm_2.7284	dimethylamine	0.649	ppm_2.7304-ppm_2.7214	dimethylamine	0.649	ppm_2.7304-ppm_2.7214	dimethylamine
Glucose	0.990	ppm_3.4624-ppm_3.4584	glucose	0.990	ppm_3.4724-ppm_3.4674	glucose	0.989	ppm_3.4884-ppm_3.4834	glucose
Glutamine	0.873	ppm_2.4674-ppm_2.4654	glutamine, 3-hydroxyisobutyrate, carnitine	0.870	ppm_2.4554-ppm_2.4524	glutamine, 3-hydroxyisobutyrate, carnitine	0.905	ppm_2.4684-ppm_2.4514	glutamine, 3-hydroxyisobutyrate, carnitine
Glycine	0.838	ppm_3.5714-ppm_3.5654	glycine, 3-hydroxyisobutyrate	0.808	ppm_3.5694-ppm_3.5674	glycine, 3-hydroxyisobutyrate	0.741	ppm_3.5704-ppm_3.5604	glycine, 3-hydroxyisobutyrate
Histidine	0.548	ppm_2.1444-ppm_2.1424	glutamate, glutamine	0.764	ppm_7.0894-ppm_7.0504	histidine	0.523	ppm_2.1514-ppm_2.1284	glutamate, glutamine
Isobutyrate	0.845	ppm_1.0724-ppm_1.0704	isobutyrate, 3-hydroxyisobutyrate	0.793	ppm_1.0854-ppm_1.0824	isobutyrate, 3-hydroxyisobutyrate	0.568	ppm_1.0854-ppm_1.0744	isobutyrate, 3-hydroxyisobutyrate
Isoleucine	0.866	ppm_1.0104-ppm_1.0084	isoleucine	0.911	ppm_1.0224-ppm_1.0204	isoleucine	0.808	ppm_1.0554-ppm_1.0524	valine
Lactate	0.988	ppm_4.1294-ppm_4.1274	lactate	0.989	ppm_4.1184-ppm_4.1164	lactate	0.989	ppm_4.1294-ppm_4.1154	lactate
Phenylalanine	0.850	ppm_7.3424-ppm_7.3394	phenylalanine	0.874	ppm_7.3914-ppm_7.3874	phenylalanine	0.888	ppm_7.4344-ppm_7.4294	phenylalanine
Proline	0.754	ppm_2.3434-ppm_2.3384	proline, glutamate	0.937	ppm_4.1524-ppm_4.1504	proline	0.699	ppm_3.3504-ppm_3.3344	proline, glutamate
Pyruvate	0.884	ppm_2.3814-ppm_2.3794	pyruvate, 3-hydroxyisovalerate, succinate	0.968	ppm_2.3794-ppm_2.3774	pyruvate, 3-hydroxyisovalerate, succinate	0.941	ppm_2.3794-ppm_2.3744	pyruvate, proline, glutamate
Threonine	0.494	ppm_2.1444-ppm_2.1424	glutamate, glutamine	0.502	ppm_6.9104-ppm_6.9074	tyrosine	0.490	ppm_6.9114-ppm_6.8934	tyrosine
Trimethylamine N-Oxide	0.282	ppm_3.2814-ppm_3.2694	trimethylamine-N-oxide, glucose, betaine, myo-inositol	0.401	ppm_3.2784-ppm_3.2704	trimethylamine-N-oxide, glucose, betaine, myo-inositol	0.403	ppm_3.2784-ppm_3.2724	trimethylamine-N-oxide, glucose, betaine, myo-inositol
Tyrosine	0.931	ppm_6.9004-ppm_6.8854	tyrosine	0.941	ppm_7.1924-ppm_7.1894	tyrosine	0.947	ppm_6.9114-ppm_6.8934	tyrosine
Valine	0.811	ppm_0.9674-ppm_0.9654	leucine, macromolecule	0.947	ppm_1.0544-ppm_1.0524	valine	0.961	ppm_1.0434-ppm_1.0404	valine

	JBA			SRV			EB (0.01 ppm)			EB (0.02 ppm)		
	correlation coef.	spectral position	metabolite	correlation coef.	spectral position	metabolite	correlation coef.	spectral position	metabolite	correlation coef.	spectral position	metabolite
3-HB	0.768	2.3964	3-HB, carnitine, glutamate	0.600	ppm_2.3994-2.3924	3-HB, carnitine, glutamate	0.547	ppm_2.395	3-HB, carnitine, glutamate	0.498	ppm_2.33	glutamate, proline
Acetate	0.966	1.9254	acetate	0.908	ppm_1.9344-1.9184	acetate	0.946	ppm_1.925	acetate	0.892	ppm_1.93	acetate
Acetoacetate	0.670	2.3804	3-hydroxyisovalerate, pyruvate, succinate	0.611	ppm_6.2884-6.2754	noise	0.614	ppm_6.285	noise	0.603	ppm_6.29	noise
Acetone	0.530	2.4324	glutamine, 3-hydroxyisobutyrate, carnitine	0.472	ppm_2.4344-2.4294	glutamine, 3-HB, 3-hydroxyisobutyrate, carnitine	0.455	ppm_2.235	acetone, macromolecules	0.350	ppm_2.33	2-hydroxyglutarate, glutamate, proline
Alanine	0.722	1.4859	alanine	0.915	ppm_1.5054-1.4704	alanine	0.926	ppm_1.495	alanine	0.905	ppm_1.49	alanine
Asparagine	0.563	2.4559	glutamine, 3-hydroxyisobutyrate, carnitine	0.698	ppm_2.1474-2.1414	glutamate, glutamine	0.683	ppm_2.145	glutamate, glutamine	0.659	ppm_2.15	glutamate, glutamine
Betaine	0.689	3.2724	glucose, betaine, myo-inositol, trimethylamine-N-oxide	0.481	ppm_3.9104-3.9064	glucose, betaine	0.630	ppm_3.275	glucose, betaine, myo-inositol, trimethylamine-N-oxide	0.221	ppm_4.07	creatinine, myo-inositol
Carnitine	0.692	2.4464	glutamine, carnitine	0.412	ppm_1.8284-1.8164	noise	0.444	ppm_3.045	creatine, creatinine	0.445	ppm_3.05	creatine, creatinine
Creatine	0.909	3.9379	creatine	0.842	ppm_3.0454-3.0384	creatine	0.763	ppm_3.935	creatine	0.626	ppm_3.05	creatine, creatinine
Creatinine	0.443	4.0704	creatinine, myo-inositol	0.749	ppm_4.0654-4.0504	creatinine	0.703	ppm_4.065	creatinine, myo-inositol	0.619	ppm_4.07	creatinine, myo-inositol
Dimethylamine	0.598	1.7729	noise, macromolecules	0.684	ppm_2.7324-2.7244	dimethylamine	0.587	ppm_1.765	noise, macromolecules	0.592	ppm_1.77	noise, macromolecules
Glucose	0.987	3.4599	glucose	0.989	ppm_3.4924-3.4774	glucose	0.990	ppm_3.475	glucose	0.988	ppm_3.47	glucose
Glutamine	0.612	2.4559	glutamine, 3-hydroxyisobutyrate, carnitine	0.779	ppm_2.1474-2.1414	glutamate, glutamine	0.897	ppm_2.455	glutamine, 3-hydroxyisobutyrate, carnitine	0.878	ppm_2.45	glutamine, 3-hydroxyisobutyrate, carnitine
Glycine	0.332	3.0579	creatine, creatinine	0.778	ppm_3.5724-3.5634	glycine, 3-hydroxyisobutyrate	0.655	ppm_3.565	glycine, 3-hydroxyisobutyrate	0.543	ppm_3.57	glycine, 3-hydroxyisobutyrate
Histidine	0.453	2.4559	glutamine	0.571	ppm_2.1474-2.1414	glutamate, glutamine	0.558	ppm_2.145	glutamate, glutamine	0.631	ppm_7.07	histidine
Isobutyrate	0.445	1.7244	leucine	0.687	ppm_1.0734-1.0664	isobutyrate, 3-hydroxyisobutyrate	0.618	ppm_1.085	isobutyrate, 3-hydroxyisobutyrate	0.522	ppm_1.05	valine
Isoleucine	0.735	0.9724	macromolecules, unknown (?), leucine	0.762	ppm_1.0594-1.0314	valine	0.792	ppm_1.045	valine	0.790	ppm_1.05	valine
Lactate	0.979	4.1204	lactate	0.983	ppm_4.1434-4.1004	lactate	0.990	ppm_4.115	lactate	0.985	ppm_4.11	lactate
Phenylalanine	0.819	7.3424	phenylalanine	0.818	ppm_7.4394-7.4124	phenylalanine	0.810	ppm_7.385	phenylalanine	0.800	ppm_7.39	phenylalanine
Proline	0.676	3.3434	proline, unknown (?)	0.871	ppm_4.1534-4.1484	proline	0.680	ppm_3.345	proline, xylose (?)	0.609	ppm_3.35	proline, xylose (?)
Pyruvate	0.884	2.3804	pyruvate, 3-hydroxyisovalerate, succinate	0.931	ppm_2.3824-2.3734	pyruvate, 3-hydroxyisovalerate, succinate	0.857	ppm_2.375	pyruvate, 3-hydroxyisovalerate, succinate	0.692	ppm_6.27	noise
Threonine	0.426	7.2014	tyrosine	0.472	ppm_6.9174-6.8864	tyrosine	0.490	ppm_6.895	tyrosine	0.474	ppm_6.89	tyrosine
Trimethylamine N-Oxide	0.449	3.2724	trimethylamine-N-oxide, glucose, betaine, myo-inositol	0.279	ppm_3.1614-3.1554	dimetyl sulfone (?), Ca-EDTA2+	0.400	ppm_3.275	trimethylamine-N-oxide, glucose, betaine, myo-inositol	0.231	ppm_8.09	noise
Tyrosine	0.816	7.1944	tyrosine	0.935	ppm_6.9174-6.8864	tyrosine	0.939	ppm_6.895	tyrosine	0.924	ppm_6.91	tyrosine
Valine	0.725	0.9724	leucine, macromolecule	0.924	ppm_1.0594-1.0314	valine	0.952	ppm_1.055	valine	0.952	ppm_1.05	valine

Table S2: Number of metabolite features extracted from 1D ^{1}H NMR spectra of (a) urinary and (b) plasma AKI data sets for different binning approaches. ^{a}Number of bins after exclusion of 21 bins corresponding to filter residues and free EDTA. ^{b}Number of bins after exclusion of 16 bins corresponding to filter residues and free EDTA. ^{c}Number of bins after exclusion of 29 bins corresponding to filter residues and free EDTA. ^{d}Number of bins after exclusion of 12 bins corresponding to filter residues and free EDTA. ^{e}Number of bins after exclusion of 28 bins corresponding to filter residues and free EDTA. ^{f}Number of bins after exclusion of 12 bins corresponding to filter residues and free EDTA. ^{g}Number of bins after exclusion of 20 bins corresponding to filter residues and free EDTA. ^{h}Number of bins after exclusion of 36 bins corresponding to filter residues and free EDTA. ^{i}Number of bins after exclusion of 28 bins corresponding to filter residues and free EDTA. ^{j}Number of bins after exclusion of 15 bins corresponding to filter residues and free EDTA.

(a) Urinary AKI data set.

	BF (λ = 1) plateaus	BF (λ = 1) non-plateaus	BF (λ = 2.5) plateaus	BF (λ = 2.5) non-plateaus	BF (λ = 5) plateaus	BF (λ = 5) non-plateaus	SRV	JBA	EB (0.01 ppm)	EB (0.02 ppm)
No. regions	180	268	498	383	574	309	585	731	700	350

(b) Plasma AKI data set.

	BF (λ = 1) plateaus	BF (λ = 1) non-plateaus	BF (λ = 2.5) plateaus	BF (λ = 2.5) non-plateaus	BF (λ = 5) plateaus	BF (λ = 5) non-plateaus	SRV	JBA	EB (0.01 ppm)	EB (0.02 ppm)
No. regions	333^a	273^b	503^c	249^d	723^e	224^f	533^g	520^h	712^i	355^j

4 Supplementary Figures

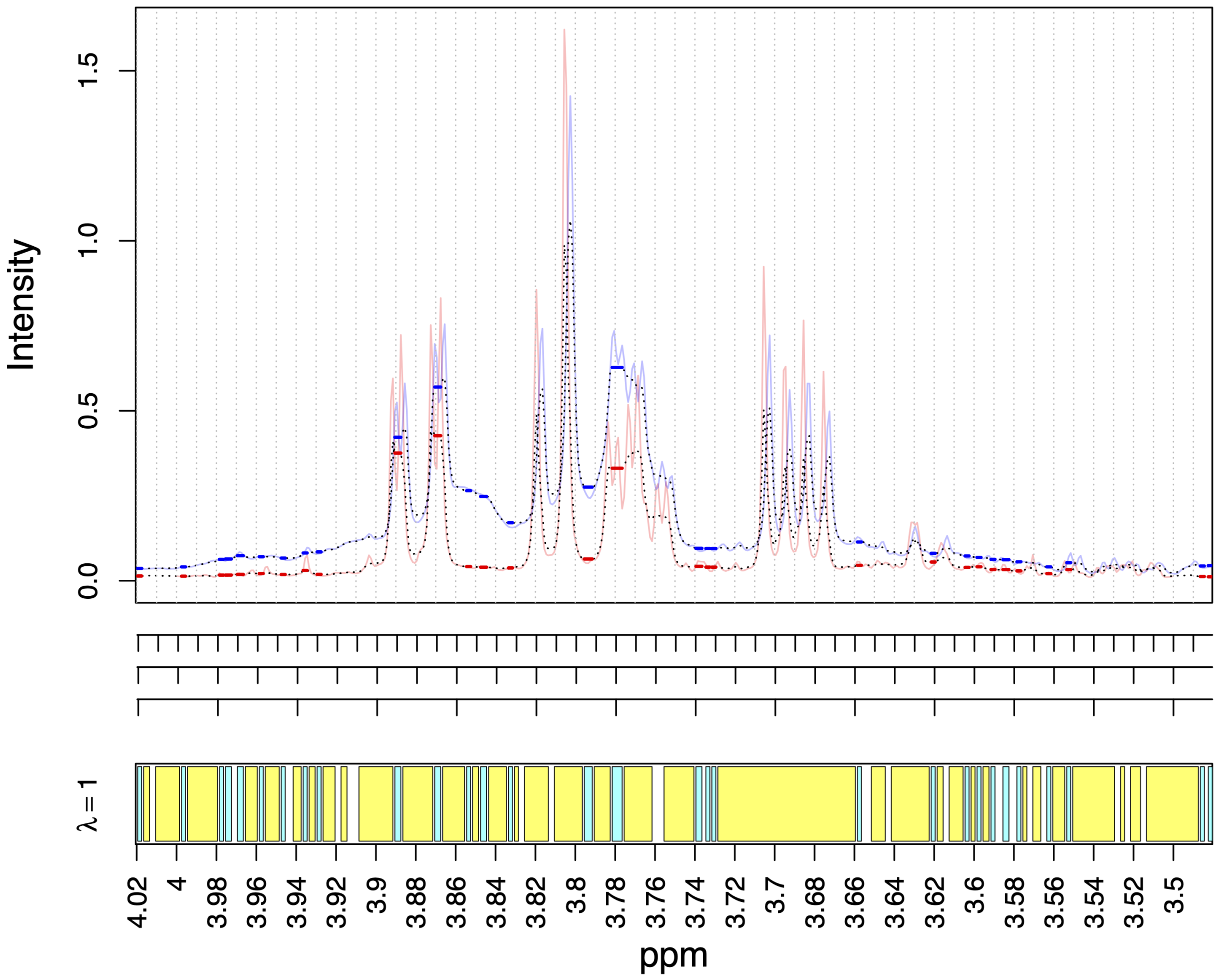

Figure S1: Exemplary NMR spectral regions ranging from 4 ppm to 3.5 ppm together with their corresponding BF fits for $\lambda = 1$. The Figure can be understood analogous to Figure 1a and b in the main article.

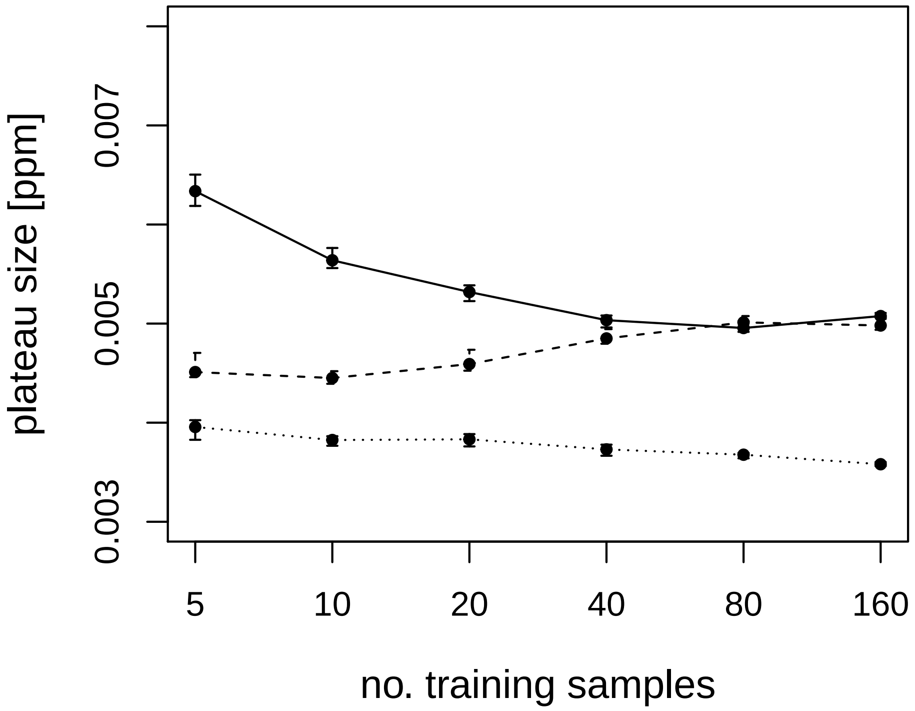

Figure S2: Average plateau widths versus the number of training samples for $\lambda = 1$ (dotted line), $\lambda = 2.5$ (dashed line), $\lambda = 5$ (solid line) in the GCKD data set. The error bars indicate the 25% to 75% percentiles and the dots the corresponding median values across 50 simulation runs.

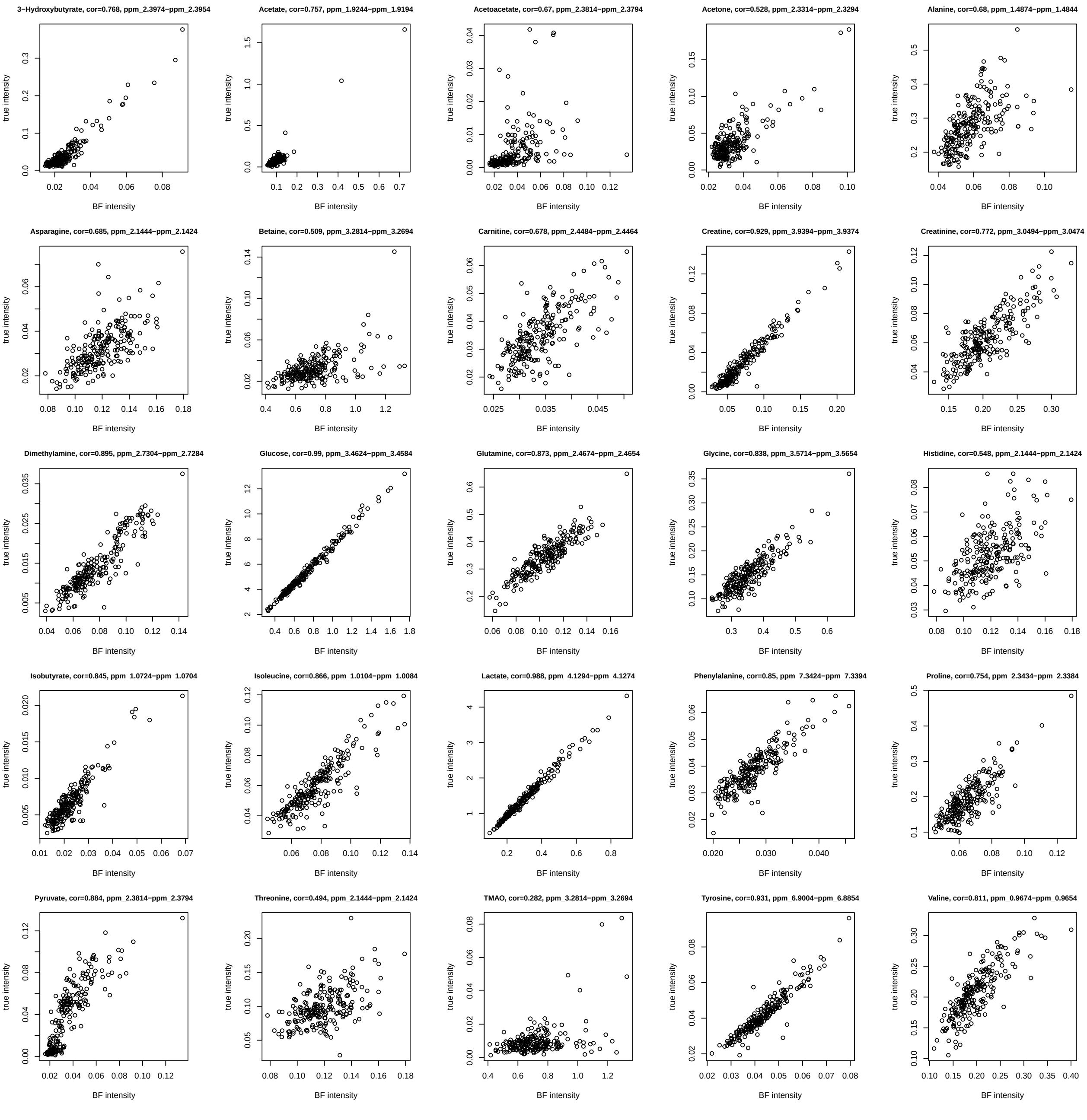

Figure S3: Comparison of the integrals of spectral features constructed by the Bucket Fuser (BF) using $\lambda = 1$ with absolutely quantified metabolite concentrations for 25 metabolites. The x-axis gives the spectral intensities returned by the Bucket Fuser which correlate best with the absolute concentrations shown on the y-axis. The headings indicate the investigated metabolites, followed by Spearman's correlation, and the selected spectral region from the BF binning in ppm. Abbr.: TMAO, trimethylamine-N-oxide.

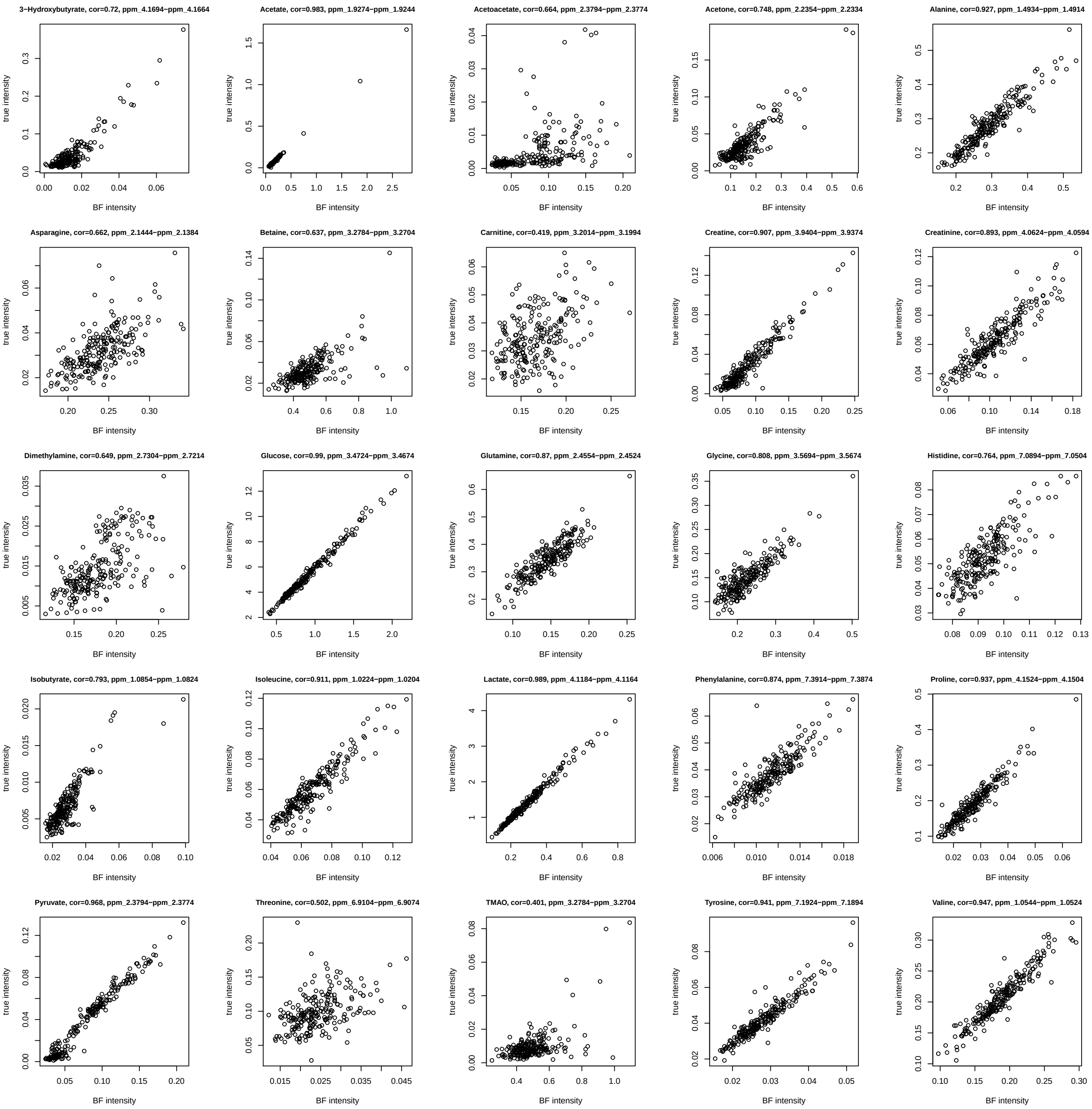

Figure S4: Comparison of the integrals of spectral features constructed by the Bucket Fuser (BF) using $\lambda = 2.5$ with absolutely quantified metabolite concentrations for 25 metabolites. The x-axis gives the spectral intensities returned by the Bucket Fuser which correlate best with the absolute concentrations shown on the y-axis. The headings indicate the investigated metabolites, followed by Spearman's correlation, and the selected spectral region from the BF binning in ppm. Abbr.: TMAO, trimethylamine-N-oxide.

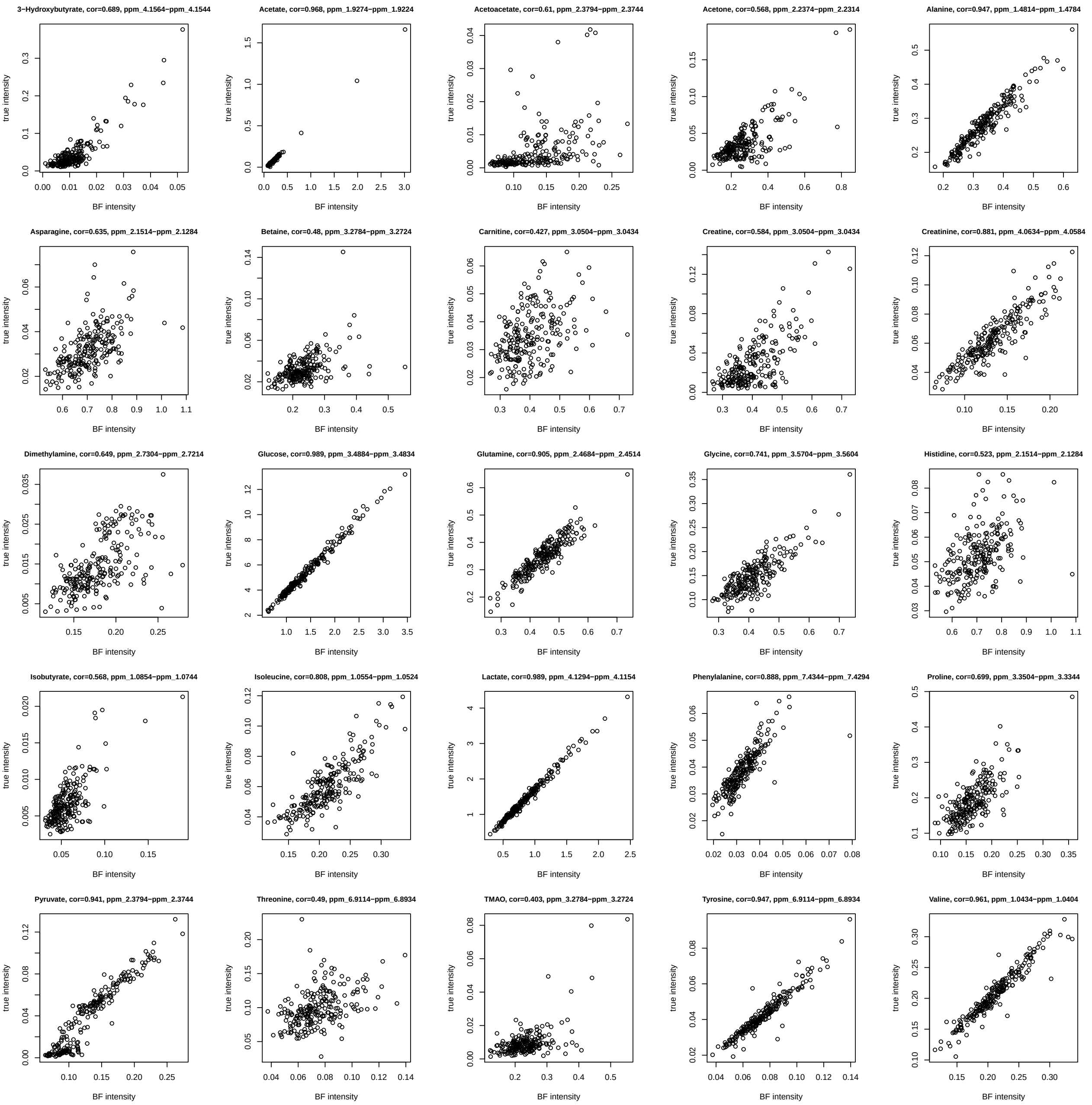

Figure S5: Comparison of the integrals of spectral features constructed by the Bucket Fuser (BF) using $\lambda = 5$ with absolutely quantified metabolite concentrations for 25 metabolites. The x-axis gives the spectral intensities returned by the Bucket Fuser which correlate best with the absolute concentrations shown on the y-axis. The headings indicate the investigated metabolites, followed by Spearman's correlation, and the selected spectral region from the BF binning in ppm. Abbr.: TMAO, trimethylamine-N-oxide.

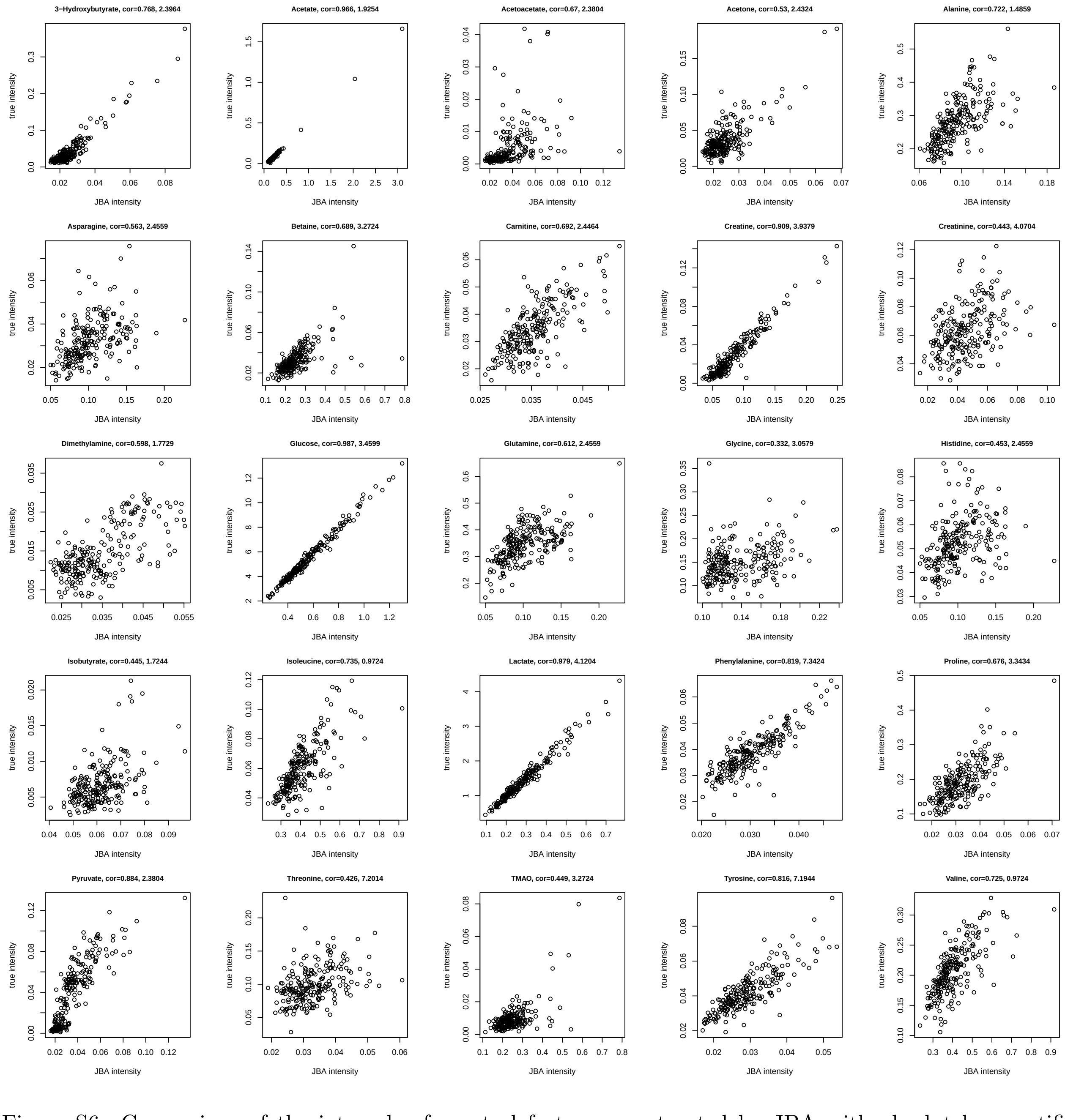

Figure S6: Comparison of the integrals of spectral features constructed by JBA with absolutely quantified metabolite concentrations for 25 metabolites. The x-axis gives the spectral intensities returned by JBA which correlate best with the absolute concentrations shown on the y-axis. The headings indicate the investigated metabolites, followed by Spearman's correlation, and the selected spectral region in ppm. Abbr.: TMAO, trimethylamine-N-oxide.

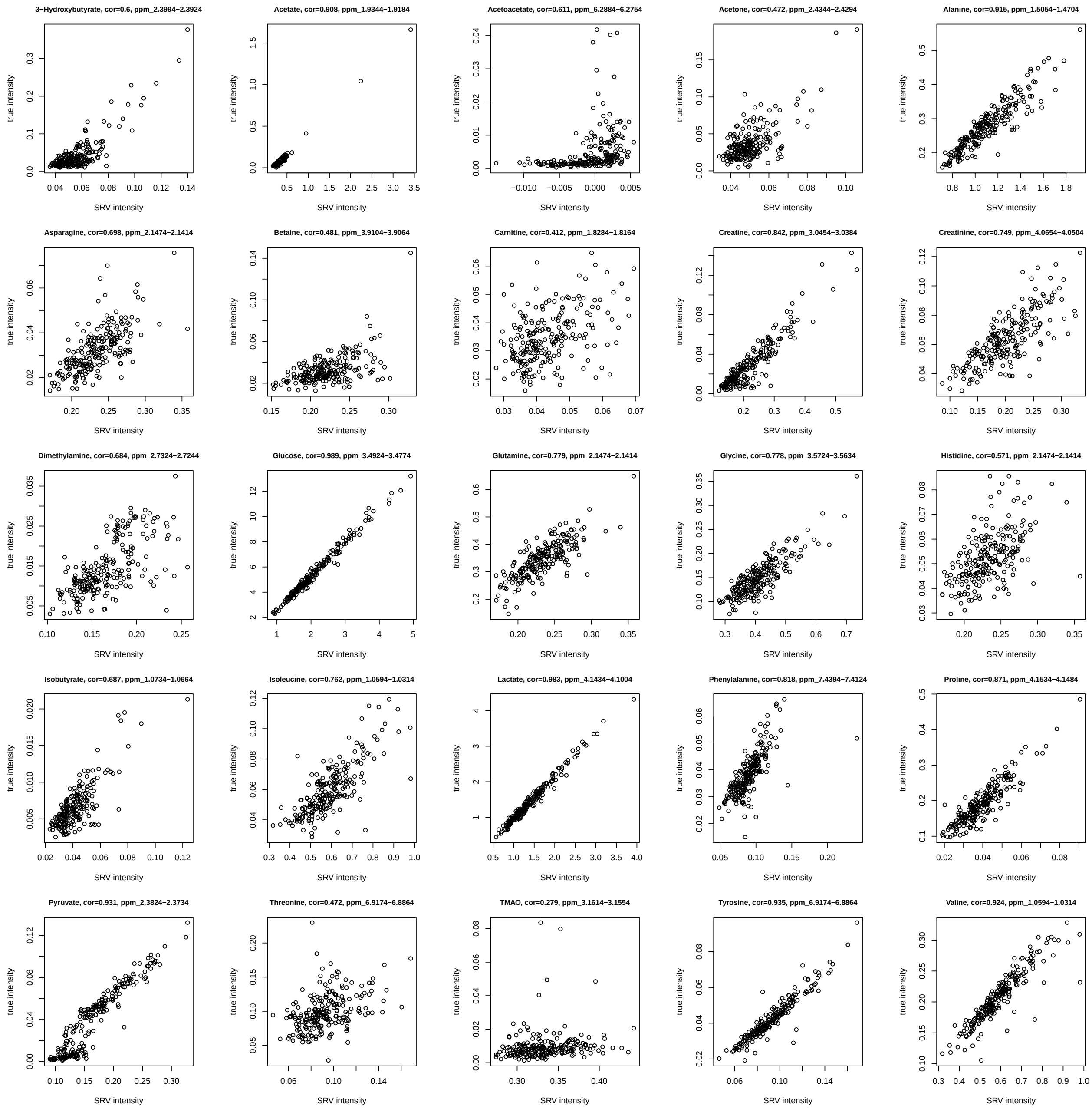

Figure S7: Comparison of the integrals of spectral features constructed by SRV with absolutely quantified metabolite concentrations for 25 metabolites. The x-axis gives the spectral intensities returned by the Bucket Fuser which correlate best with the absolute concentrations shown on the y-axis. The headings indicate the investigated metabolites, followed by Spearman's correlation, and the selected spectral region from the BF binning in ppm. Abbr.: TMAO, trimethylamine-N-oxide.

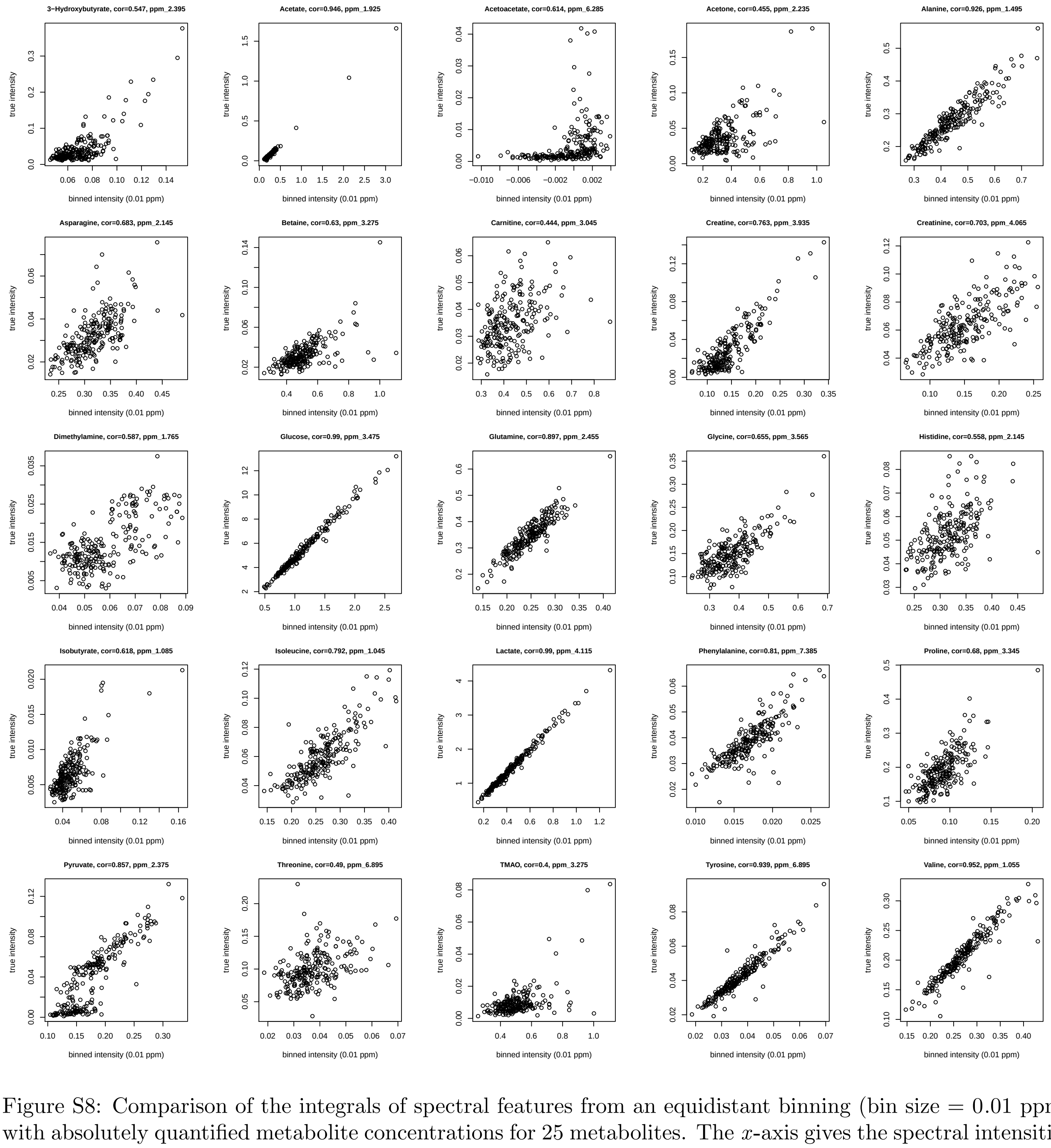

Figure S8: Comparison of the integrals of spectral features from an equidistant binning (bin size = 0.01 ppm) with absolutely quantified metabolite concentrations for 25 metabolites. The x-axis gives the spectral intensities returned by the binning which correlate best with the absolute concentrations shown on the y-axis. The headings indicate the investigated metabolites, followed by Spearman's correlation, and the selected spectral region in ppm. Abbr.: TMAO, trimethylamine-N-oxide.

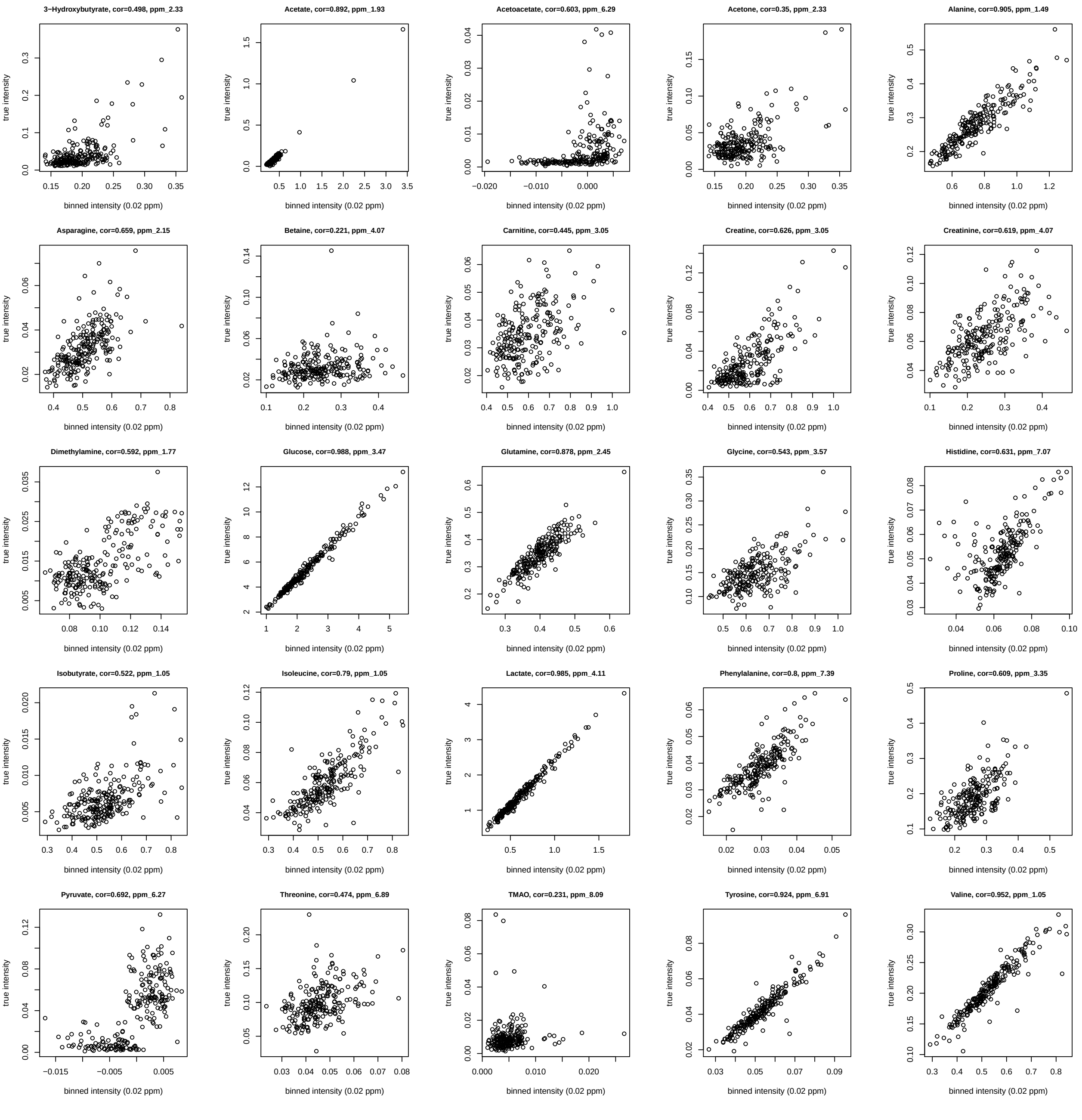

Figure S9: Comparison of the integrals of spectral features from an equidistant binning (bin size = 0.02 ppm) with absolutely quantified metabolite concentrations for 25 metabolites. The x-axis gives the spectral intensities returned by the binning which correlate best with the absolute concentrations shown on the y-axis. The headings indicate the investigated metabolites, followed by Spearman's correlation, and the selected spectral region in ppm. Abbr.: TMAO, trimethylamine-N-oxide.

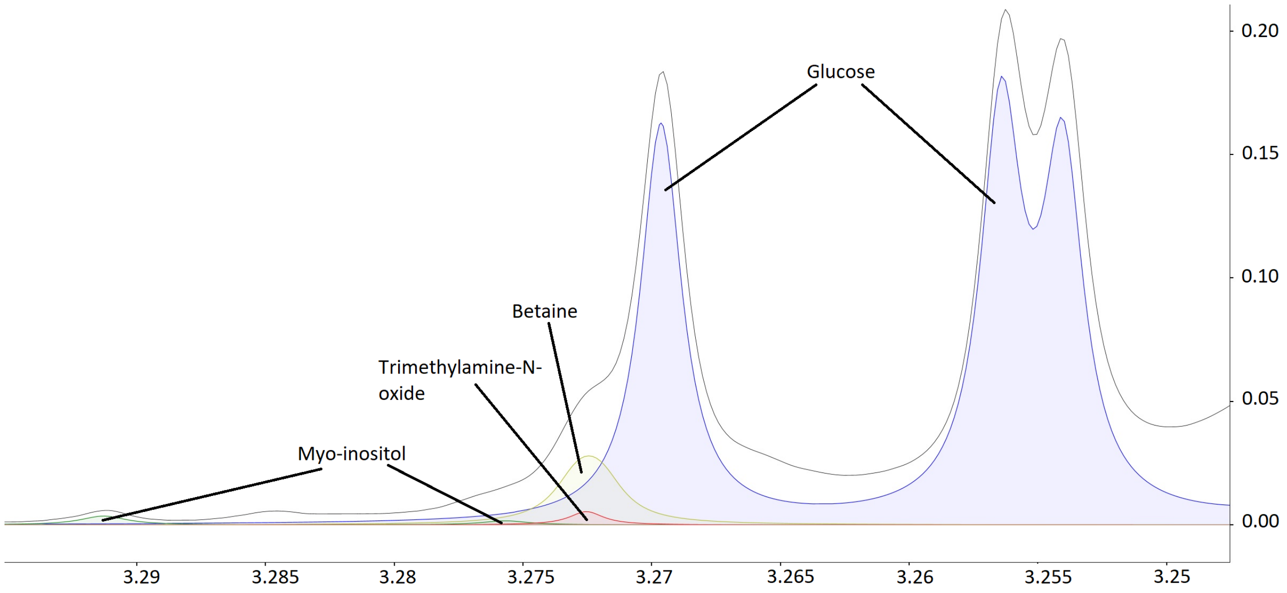

Figure S10: Exemplary NMR spectral region from 3.29 to 3.25 ppm, comprising metabolite signals of D-glucose, betaine, trimethylamine-N-oxide, and myo-inositol. One exemplary GCKD NMR spectrum is shown in black, reference spectra of the pure compounds D-glucose, betaine, trimethylamine-N-oxide, and myo-inositol manually fitted to the GCKD NMR spectrum, are shown in blue, light green, red, and dark green, respectively.

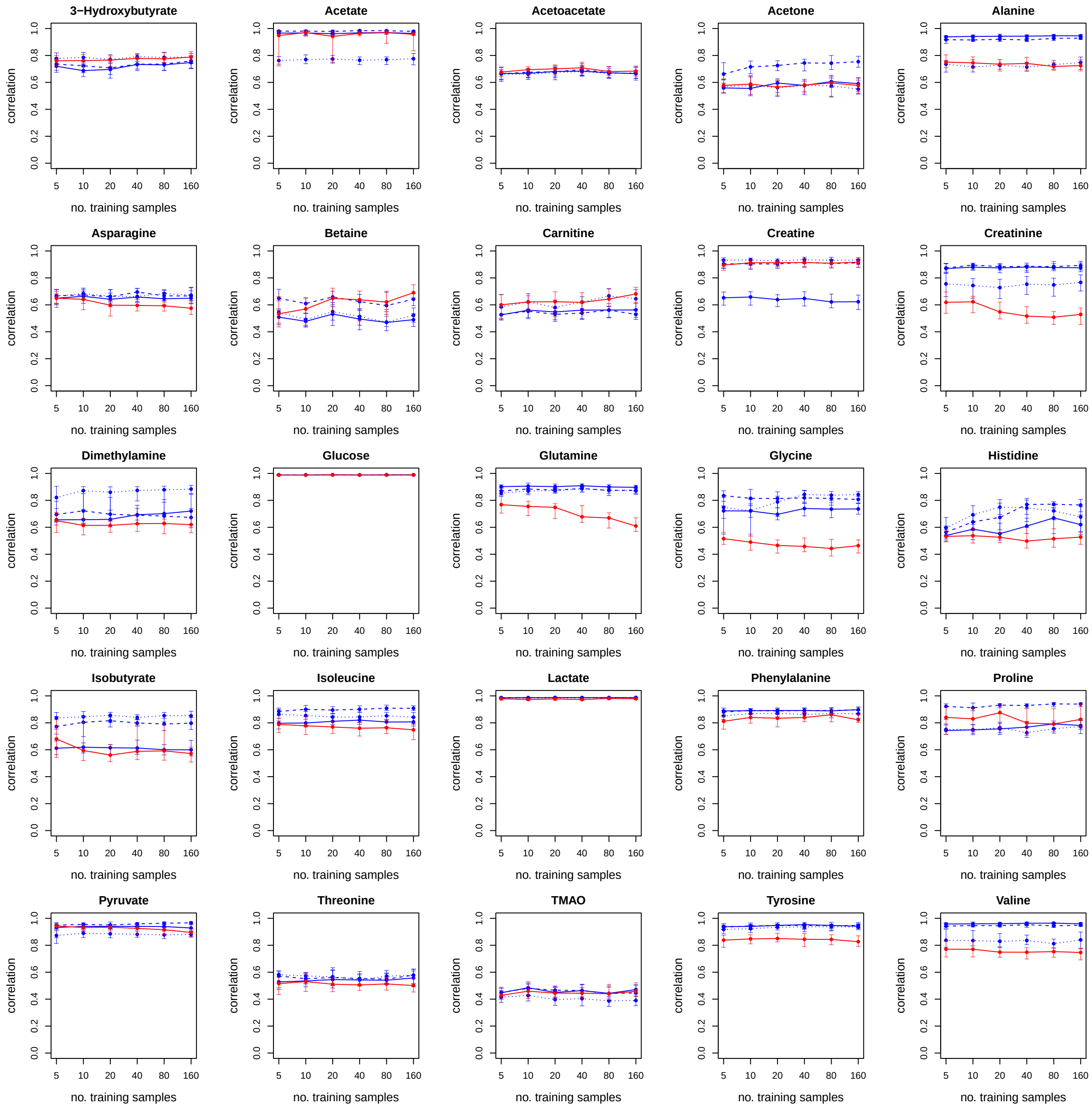

Figure S11: Spearman's correlations between absolutely quantified metabolite concentrations and integrals of corresponding spectral features from different binning approaches in dependence of the number of training samples n for 25 absolutely quantified metabolites in the GCKD data set. The points give the median Spearman's correlation obtained on test data and the whiskers the corresponding 25% and 75% quartiles. The dotted, dashed, and solid blue lines correspond to BF with $\lambda = 1$, $\lambda = 2.5$, and $\lambda = 5$, respectively, the solid red line to JBA. The metabolite identity is given in the header of the plots. Abbr.: TMAO, trimethylamine-N-oxide.

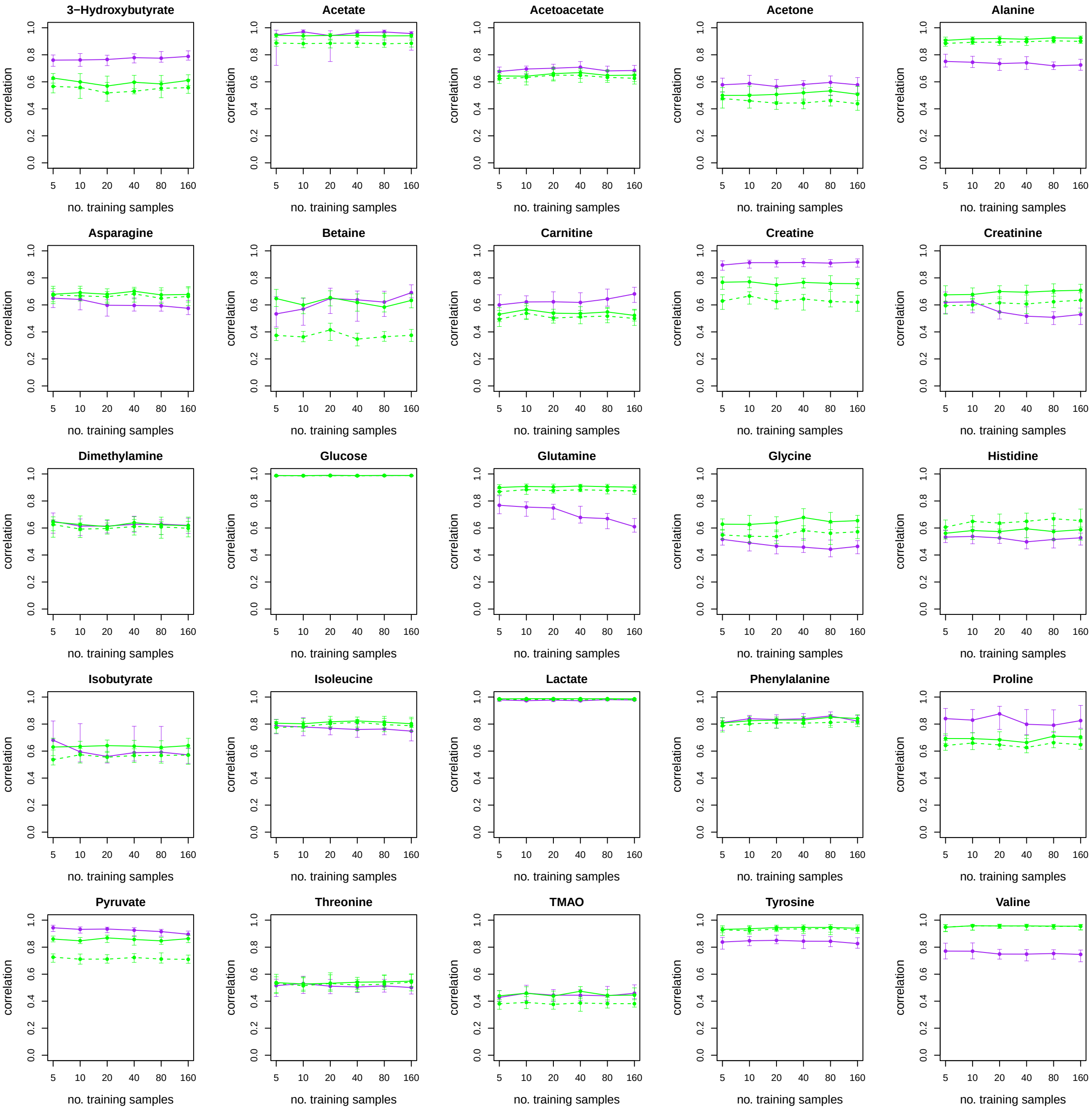

Figure S12: Spearman's correlations between absolutely quantified metabolite concentrations and integrals of corresponding spectral features from different binning approaches in dependence of the number of training samples n for 25 absolutely quantified metabolites in the GCKD data set. The points give the median Spearman's correlation obtained on test data and the whiskers the corresponding 25% and 75% quartiles. The solid purple lines correspond to SRV and the green solid and dashed lines to an equidistant binning with bin size 0.01 ppm and 0.02 ppm, respectively. The metabolite identity is given in the header of the plots. Abbr.: TMAO, trimethylamine-N-oxide.

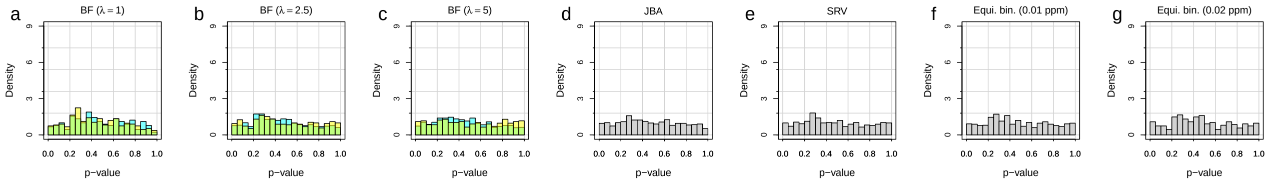

Figure S13: P-value distributions for the comparison AKI versus non-AKI after cardiac surgery based on permuted urine data. Figure (a) to (g) correspond to the different binning approaches, BF with $\lambda = 1$, BF with $\lambda = 2.5$, BF with $\lambda = 5$, JBA, SRV, equidistant binning with bin size 0.01 ppm, and equidistant binning with bin size 0.02 ppm, respectively. For the BF method, the same color coding as, e.g., in Figure 1 was applied. Please note that light green bars correspond to the overlap of cyan and yellow bars.

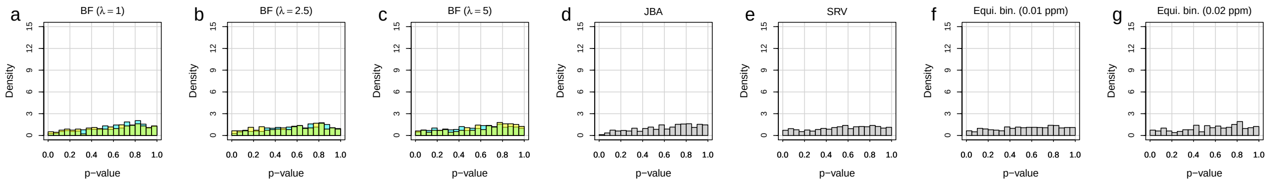

Figure S14: P-value distributions for the comparison AKI versus non-AKI after cardiac surgery based on permuted plasma data. Figure (a) to (g) correspond to the different binning approaches, BF with $\lambda = 1$, BF with $\lambda = 2.5$, BF with $\lambda = 5$, JBA, SRV, equidistant binning with bin size 0.01 ppm, and equidistant binning with bin size 0.02 ppm, respectively. For the BF method, the same color coding as, e.g., in Figure 1 was applied. Please note that light green bars correspond to the overlap of cyan and yellow bars.

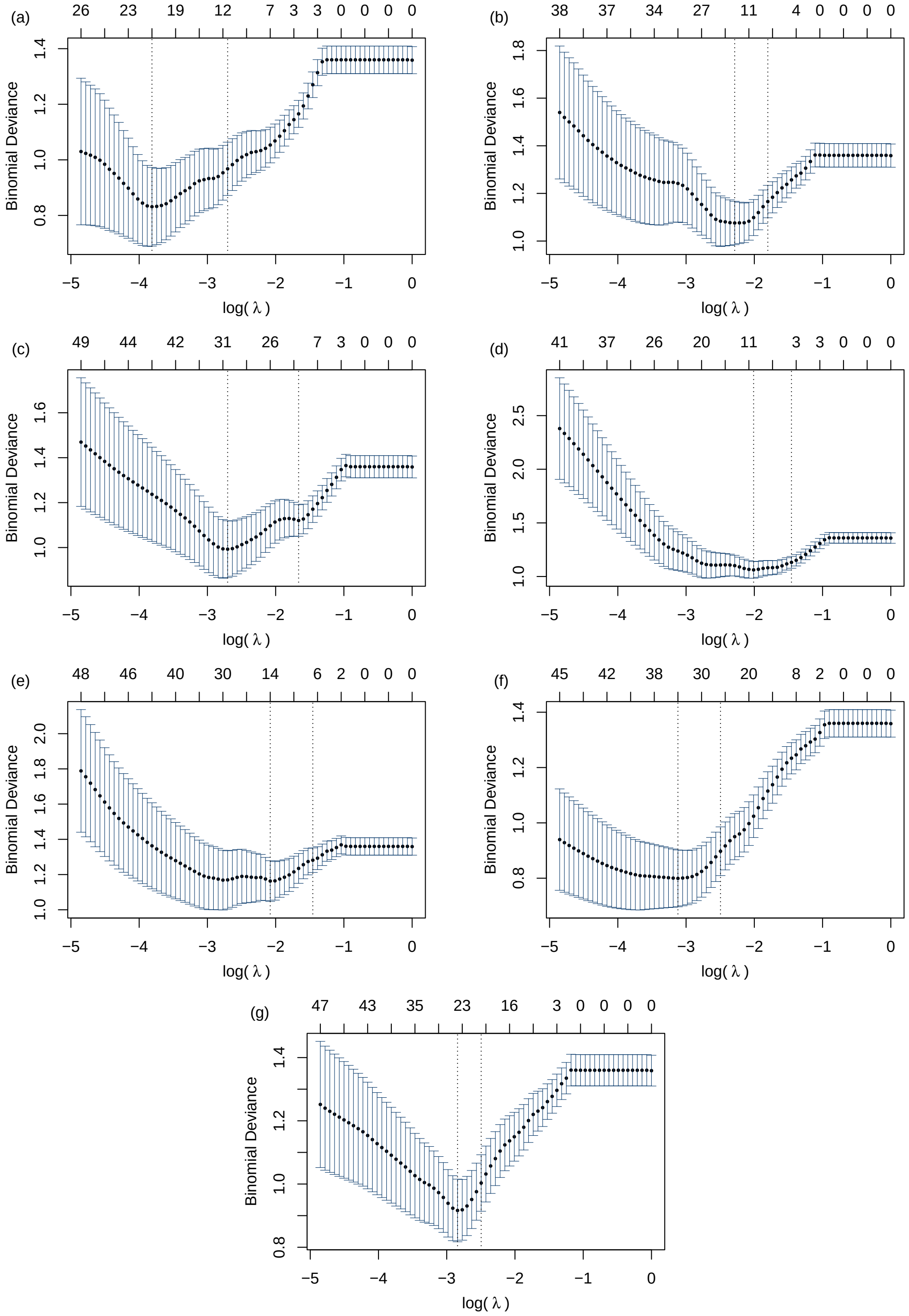

Figure S15: Hyper-parameter calibration of binomial zero-sum regression for the plasma AKI data set. Figures correspond to BF ($\lambda = 1$) (a), BF ($\lambda = 2.5$) (b), BF ($\lambda = 5$) (c), JBA (d), SRV (e), EB (0.01 ppm) (f), and EB (0.02 ppm) (g). The x-axis gives $\log(\lambda)$ corresponding to the size of the l_1 regularization term of zero-sum regression (not to be confused with the BF regularization) and the y-axis gives the binomial deviance in a leave-one-out cross validation. The values at the top of the figures give the number of selected NMR features.

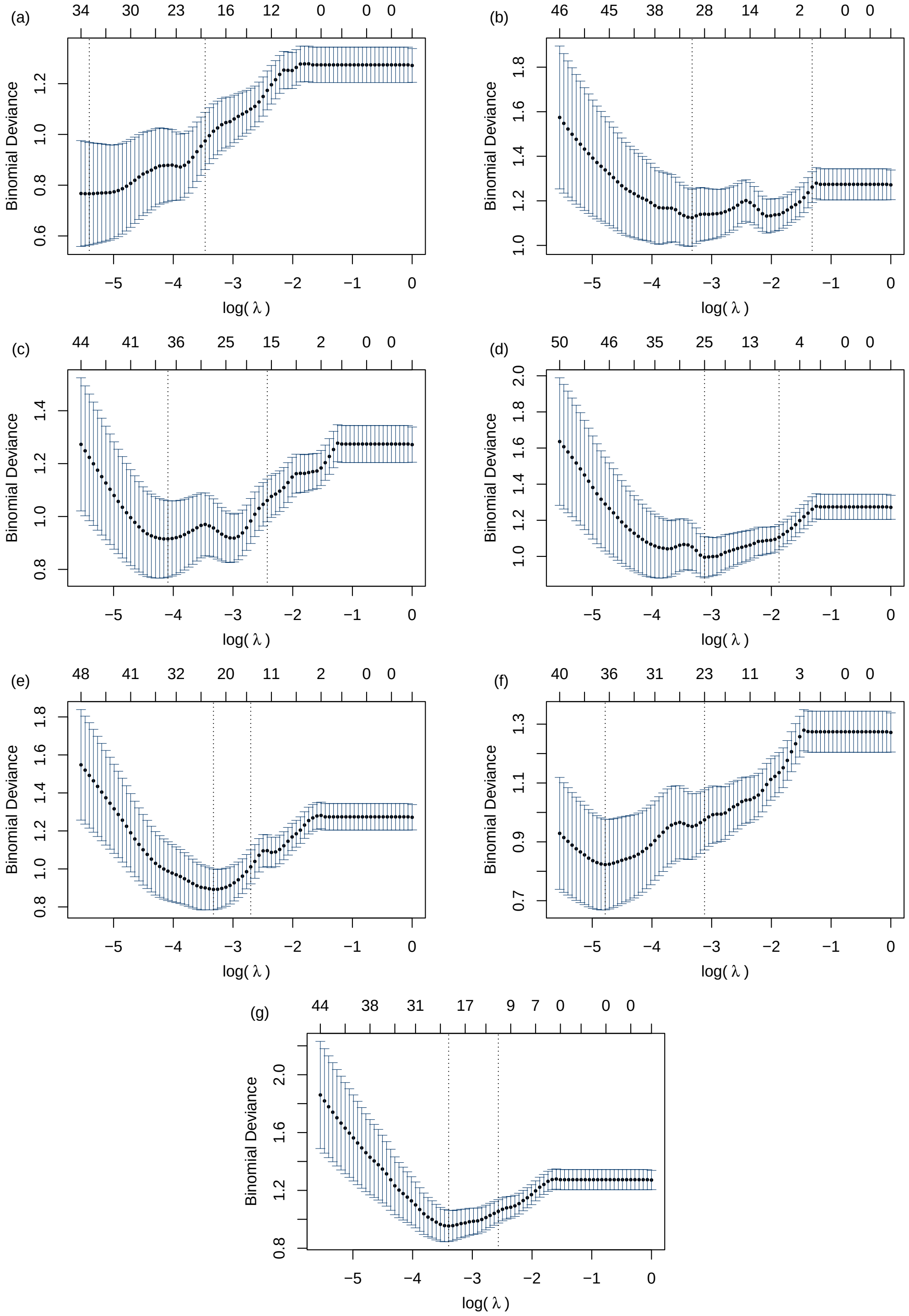

Figure S16: Hyper-parameter calibration of binomial zero-sum regression for the urinary AKI data set. Figures correspond to BF ($\lambda = 1$) (a), BF ($\lambda = 2.5$) (b), BF ($\lambda = 5$) (c), JBA (d), SRV (e), EB (0.01 ppm) (f), and EB (0.02 ppm) (g). The x-axis gives $\log(\lambda)$ corresponding to the size of the l_1 regularization term of zero-sum regression (not to be confused with the BF regularization) and the y-axis gives the binomial deviance in a leave-one-out cross validation. The values at the top of the figures give the number of selected NMR features.

5 Relationship between the choice of logarithmic base and the regularization parameter λ

Data values on a logarithmic scale with base b can be translated to base b' by

$$\log_b(y_{ij}) = \frac{\log_{b'}(y_{ij})}{\log_{b'}(b)} .$$

With $Y_{ij} = \log_b(y_{ij})$, $Y'_{ij} = \log_{b'}(y_{ij})$ and $c = \log_{b'}(b)$, we obtain

$$Y_{ij} = \frac{1}{\log_{b'}(b)} Y'_{ij} = \frac{1}{c} Y'_{ij} .$$

Therefore, Eq. (1) from the main manuscript becomes

$$\begin{aligned}
\hat{B} &= \arg\min_B \left\{ ||Y - B||_F^2 + \lambda\sqrt{n} \sum_{j=1}^{p-1} ||B_{\cdot j} - B_{\cdot j+1}||_2 \right\} \\
&= \arg\min_B \left\{ ||\frac{1}{c}Y' - B||_F^2 + \lambda\sqrt{n} \sum_{j=1}^{p-1} ||B_{\cdot j} - B_{\cdot j+1}||_2 \right\} \\
&= \arg\min_B \left\{ ||Y' - cB||_F^2 + c^2\lambda\sqrt{n} \sum_{j=1}^{p-1} ||B_{\cdot j} - B_{\cdot j+1}||_2 \right\} \\
&= \arg\min_B \left\{ ||Y' - cB||_F^2 + c\lambda\sqrt{n} \sum_{j=1}^{p-1} ||cB_{\cdot j} - cB_{\cdot j+1}||_2 \right\}
\end{aligned} \tag{S1}$$

Thus, an estimate of B' which corresponds to the input data Y' can be obtained via

$$\hat{B}' = \arg\min_B \left\{ ||Y' - B'||_F^2 + \lambda'\sqrt{n} \sum_{j=1}^{p-1} ||B'_{\cdot j} - B'_{\cdot j+1}||_2 \right\} \tag{S2}$$

with $\lambda' = c\lambda = \log_{b'}(b)\lambda$. As outlined in the main manuscript, regularization values of $\lambda = \{1, 2.5, 5\}$ performed well given the specific input data (of given resolution and quality) and that the data were $\log_2$ transformed. If data are provided using a different logarithmic base, a re-adjustment of regularization parameters might be necessary. In our case this suggests $\lambda' = \log_{10}(2)\{1, 2.5, 5\} = \{0.301, 0.753, 1.505\}$ for $\log_{10}$-transformed data.

6 Convergence analyses of the BF algorithm

We confirmed the convergence of the BF algorithm for different step sizes ρ (Suppl. Fig. S17) and evaluated respective residuals between model fits (Suppl. Fig. S18) on the GCKD plasma data set. Details are summarized in the figure captions.

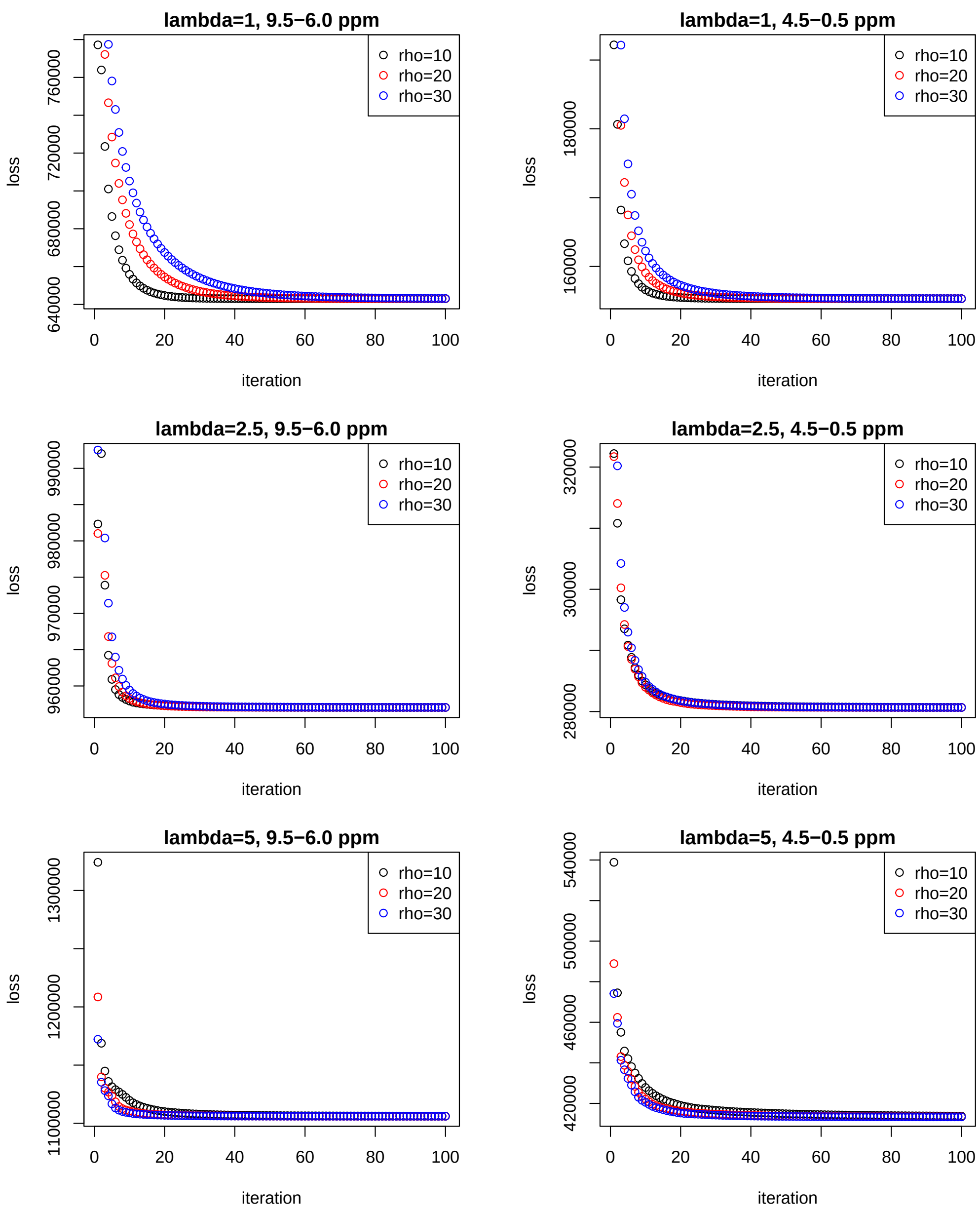

Figure S17: Losses (y-axis) versus iteration steps (x-axis). The left column corresponds to the Bucket Fuser optimization using data in the spectral region from 9.5 to 6.0 ppm and the right column to the region from 4.5 to 0.5 ppm. The rows correspond to different regularization parameters λ; $\lambda = 1$ (first row), $\lambda = 2.5$ (second row), $\lambda = 5$ (third row). The colors correspond to different step sizes ρ; $\rho = 10$ (black circles), $\rho = 20$ (red circles), and $\rho = 30$ (blue circles).

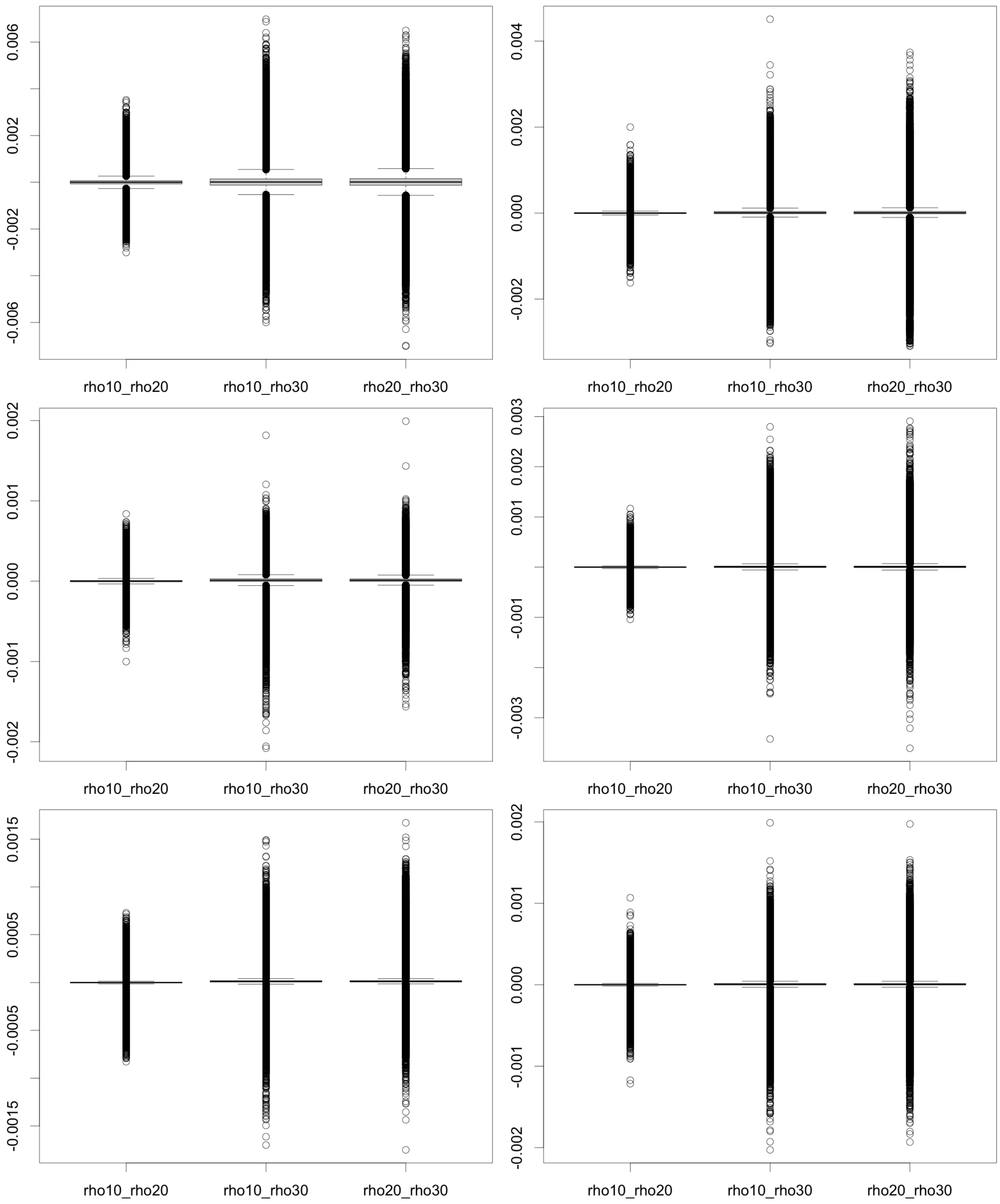

Figure S18: Residuals between different estimates of B using step sizes of $\rho \in \{10, 20, 30\}$. The sub-figures correspond to Fig. S17; input data from 9.5 to 6.0 ppm (left column), input data from 4.5 to 0.5 ppm (right column), $\lambda = 1$ (first row), $\lambda = 2.5$ (second row), $\lambda = 5$ (third row). The boxplots represent from left to right the residuals $B(\rho = 10) - B(\rho = 20)$, $B(\rho = 10) - B(\rho = 30)$, and $B(\rho = 20) - B(\rho = 30)$ in each of the subplots.
