# Supplementary material for: Bucket Fuser: Statistical Signal Extraction for 1D 1H NMR Metabolomic Data
Source: Metabolites. 2022 Aug 29;12(9):812. doi: 10.3390/metabo12090812 (PMC9501206; doi:10.3390/metabo12090812)
Supplement: Supplementary file 1 [file metabolites-12-00812-s001.zip › Supplementary_File_1.zip!/Tutorial_BF_metabolites/BF_tutorial.html]

 


BF


# Bucket Fuser example

# Contents

- Load the Bucket Fuser
- Apply the Bucket Fuser wrapper function
- Train Bucket Fuser model
- Visualization
- Bucket Fuser options
- Apply a developed Bucket Fuser model to new data

# Load the Bucket Fuser

We first load Bucket Fuser (BF) functions from `BF.py`:

```
from BF import BF, BF_aggregate_bins, apply_BF_model, BF_wrapper


import numpy as np


import pandas as pd
```

# Apply the Bucket Fuser wrapper function

We start with the BF wrapper function, which performs several successive steps. These are to (1) perform a logarithmic transformation on the NMR spectral data, (2) train a BF model on the transformed data, and (3) use the BF-derived bin boundaries to sum up the respective bin intensities using the original data. Note that the input data are assumed to be strictly positive.

```
df_original = pd.read_csv('example.csv', header=0, index_col=0)   #read example file and make a panda frame (columns: NMR regions, rows: NMR samples)


df_original_binned, BF_summary = BF_wrapper(df_original, lambda_val=2.5)


print(df_original_binned, BF_summary)   #show results
```

Here, `df_original_binned` contains the binned intensities and `BF_summary` provides a summary of the aggregated bins and whether they are derived from plateau regions. The parameter `lambda_val` defines the plateau size; larger `lambda_val` values usually give larger plateaus. Note, reasonable values for `lambda_val` were between `1` and `5` in our studies, where we used input spectra with a resolution of 0.001 ppm. Higher or lower resolved spectra might require readjusted values of `lambda_val`. However, as outlined in the main article, sample size has only a minor effect on the optimal choice of `lambda_val`.

# Train a Bucket Fuser model

Core function of the BF algorithm is to minimize Loss function (1) in the main article for given regularization parameters, i.e. `lambda_val` in our python implementation. We recommend to train the BF model on logarithmically transformed data to account for heteroscedasticity. The subsequent binning can be performed on both the original data and the log-transformed data. The BF fit for regularization parameter `lambda_val=2.5`can be performed as follows.

```
df_original = pd.read_csv('example.csv', header=0, index_col=0)   #read example file and make a panda frame (columns: NMR regions, rows: NMR samples)


df_log0 = np.log2(df_original)


df_BF0 = BF(df_log0, lambda_val=2.5)
```

This yields the model fit to the log-transformed data `df_log0`. Note, the logarithmic transformation can not deal with negative values and zeros. Here, different strategies can be used prior to logarithmic transformation. For instance, values below a given threshold can be replaced by this threshold.

Finally, the estimated BF model can be applied to the original data. Note, this yields the same data matrix as the BF wrapper function.

```
df_log_binned, df_original_binned, BF_summary = BF_aggregate_bins(df_BF0, df_original)
```

Here, `df_log_binned` is the binned BF fit (corresponding to df\_log0, i.e., data are on log2 scale), `df_original_binned` are the binned data based on the original data (as returned by the wrapper function), and `BF_summary`is the binning summary as above.

# Visualization

Visualization of previous results can be performed as follows:

```
import matplotlib.pyplot as plt


y_original_log = df_log0.iloc[0].to_numpy()#extract the log-transformed input data of the first sample


y_binned_log = df_BF0.iloc[0].to_numpy()#extract the corresponding BF model


cuts = (BF_summary['start'].iloc[1:].to_numpy() +  BF_summary['stop'].iloc[:-1].to_numpy())/2 #calculate the BF cutpoints


#for illustration purposes we focus on a small region:


x = np.arange(len(y_original_log))[2220:2350]


y_original_log = y_original_log[2220:2350]


y_binned_log = y_binned_log[2220:2350]


cuts = cuts[cuts<=np.max(x)]


cuts = cuts[cuts>=np.min(x)]


#create ppm labels:


positions = df_original.columns.tolist()


positions = [float(p.replace('ppm_', '')) for p in positions]


positions = np.array(positions)[x]


#create plot:


fig, ax = plt.subplots(figsize=(13,3))


ax.plot(x, 2**y_original_log, linewidth=2.0)    #data were translated back to their natural values


ax.plot(x, 2**y_binned_log, linewidth=2.0, color='red')    #data were translated back to their natural values


ax.set_xticks(x)


ax.set_xticklabels(np.round(positions,3), rotation=90, fontsize= 6)


for xc in cuts:


plt.axvline(x=xc, linestyle='--', linewidth =0.7)


fig.tight_layout()


plt.savefig("example_BF.png")   #save plot as png
```

This code generates the figure "example\_BF.png".

# Bucket Fuser options

The Bucket Fuser has several options to control the model fit. These are the maximum number of iterations `max_iterations` in the ADMM algorithm, `tol` is a tolerance measure, and `rho` is the stepsize of the algorithm. These options can be set in both functions `BF` and `BF_wrapper`.

```
df_original_binned, BF_summary = BF_wrapper(df_original, lambda_val=2.5, rho=10, eps=1e-12, max_iterations=10000)
```

Note, very large values of `lambda_val` require substantially more iterations to obtain reasonable fits. The function `BF_aggregate_bins` contains an option to smooth results, i.e. fits are rounded up to a given digit, i.e.,

```
BF_aggregate_bins(df_BF0, df_original, round_digits=5)
```

and results can be filtered for a minimum bin size by `min_bin_length` (the standard value is `min_bin_length = 1`, i.e., no filtering is applied)

```
BF_aggregate_bins(df_BF0, df_original, min_bin_length=3)
```

# Apply a developed Bucket Fuser model to new data

Occasionally, extracted BF boundaries need to be applied to new data, which can be performed as follows.

```
df_original_new = pd.read_csv('example_new.csv', header=0, index_col=0)


f_original_binned = apply_BF_model(df_original_new, BF_summary)
```

Here, we provided the BF summary generated by the wrapper function `BF_wrapper` or by the aggregation function `BF_aggregate_bins`.
